# Gas Sensors Based on Chemi-Resistive Hybrid Functional Nanomaterials

**DOI:** 10.1007/s40820-020-0407-5

**Published:** 2020-03-11

**Authors:** Yingying Jian, Wenwen Hu, Zhenhuan Zhao, Pengfei Cheng, Hossam Haick, Mingshui Yao, Weiwei Wu

**Affiliations:** 1 School of Advanced Materials and Nanotechnology, Interdisciplinary Research Center of Smart Sensors, Xidian University, Xi’an, 710071 People’s Republic of China; 2 School of Aerospace Science and Technology, Xidian University, Xi’an, 710071 People’s Republic of China; 3 Department of Chemical Engineering, Russell Berrie Nanotechnology Institute, Technion-Israel Institute of Technology, 3200003 Haifa, Israel; 4 Institute for Integrated Cell-Material Sciences (WPI-iCeMS), Kyoto University Institute for Advanced Study, Kyoto University, Yoshida Ushinomiya-cho, Sakyo-ku, Kyoto, 606-8501 Japan

**Keywords:** Gas sensor, Hybrid, Chemi-resistor, Functional nanomaterials

## Abstract

Chemi-resistive sensors based on hybrid functional materials are promising candidates for gas sensing with high responsivity, good selectivity, fast response/recovery, great stability/repeatability, room-working temperature, low cost, and easy-to-fabricate, for versatile applications. This progress report reviews the advantages and advances of these sensing structures compared with the single constituent, according to five main sensing forms: manipulating/constructing heterojunctions, catalytic reaction, charge transfer, charge carrier transport, molecular binding/sieving, and their combinations. Promises and challenges of the advances of each form are presented and discussed. Critical thinking and ideas regarding the orientation of the development of hybrid material-based gas sensor in the future are discussed.

## Highlights

- This review gives a thinking based on the generic mechanisms rather than simply dividing them as different types of combination of materials, which is unique and valuable for understanding and developing the novel hybrid materials in the future.
- The hybrid materials, their sensing mechanism, and their applications are systematically reviewed. Critical thinking and ideas regarding the orientation of the development of hybrid material-based gas sensor in the future are also discussed.

## Graphical abstract

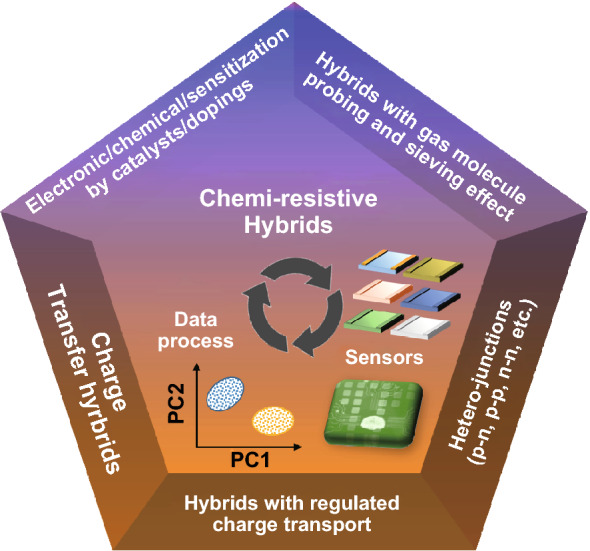

## Introduction

Monitoring and recording chemical stimulus or variations in the environment are increasingly important in future production and daily life of human health [1]. Achieving this goal relies on the availability of high-performance sensor units that are capable of detecting gas analytes, such as volatile/semi-volatile organic compounds (VOCs/SVOCs) highly rich regarding critical information for the detection, monitoring and closed-loop control in many fields, including medicine, food industry, environmental monitoring, public security, and agricultural production [2, 3].

An ideal gas sensor requires high responsivity, good selectivity, fast response/recovery, great stability/repeatability, room-working temperature, low cost, and easy-to-fabricate for practical applications [4–6]. To meet those requirements, many types of gas sensors with different transduction forms, e.g., chemi-resistor, field-effect transistor (FET), solid-state electrochemical sensor (SSES), quartz-crystal microbalance (QCM), gas capacitor, surface acoustic wave (SAW), have been well studied and developed. Among them, since the 1960s [7], a chemi-resistor that contains an active sensing layer bridging a pair of electrodes became a promising candidate due to its advantages [4–6, 8–12] including easy-to-fabricate, use of very small quantity (milligram level) active materials, wide adoption of sensitive materials, and simple sensing data, which ensure its success in certain commercialization opportunities [13–15]. However, it is rare to find chemi-resistors that can meet these specific requirements.

An emerging approach in chemi-resistors to meet these needs relies on hybrid materials, viz. materials that integrate 2+ single constituents at the nanometer or molecular level [16–36], to achieve new and/or enhanced sensing properties. In this progress report, we review the advances of the hybrid material-based gas sensors concisely and comprehensively. The hybrid materials-based chemi-resistive gas sensors are distinguished, understood, and introduced based on the generic mechanisms rather than simply dividing them as different types of combination of materials. Then, the report, in detail, focuses on the research and development (R&D) aspects of hybrid gas sensors, while presenting and discussing the sensing performances of different types of hybrid materials, and associated enhanced sensing mechanisms. Promises and challenges toward the future development of each elements are deeply thought and discussed. Critical thinking and ideas regarding the orientation of the development of hybrid material-based gas sensor in the future are also discussed.

## The Need for Hybrid Functional Nanomaterials for Sensing Applications

**Fig. 1:**
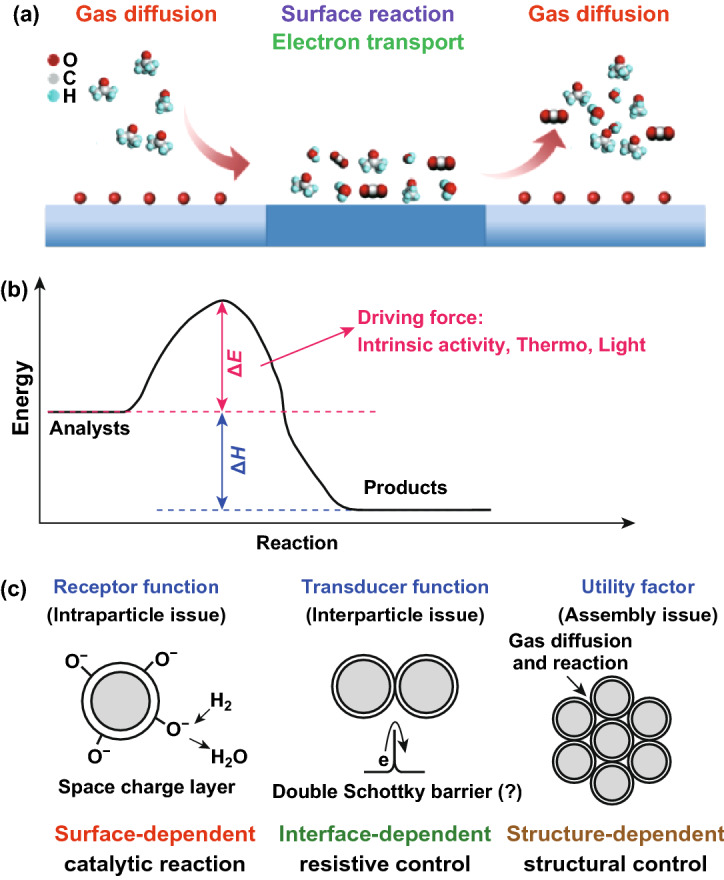
Schematic illustration of **a** three gas-sensing units and **b** sensing reactions. **c** The enhanced gas-sensing mechanisms for hybrid chemi-resistive nanomaterials. The upper part of **c** was modified from reference. Reproduced with permission [38]. Copyright 2009, Elsevier

Chemi-resistors for gas sensing include three main processes: diffusion/molecule capture unit, surface reaction unit (including charge transfer), and charge carrier transport unit (Fig. 1a) [37]. To date, most of these sensors and/or sensor arrays utilize sensing elements that are based on single material or transduction mechanism, of which intrinsic sensing activity or additional thermal/photonic energy are usually employed as the driving force to stimulate the sensing effects of target gases (Fig. 1b). The hindrances are unavoidable at several levels: (i) not satisfying long-term stability and sensitivity of organic chemi-resistors due to the high affinity of conductive polymers (CP), such as polyaniline (PANI), polypyrrole (PPy), and polythiophene (PTh), toward volatile organic compounds (VOCs) and humidity existed in the atmosphere; (ii) high operating temperatures (usually > 200 °C), baseline drift, limited selectivity, and oxidation/decomposition of VOCs in the case of inorganic materials (especially metal oxide materials, e.g., ZnO, SnO, TiO_2_, SnO_2_)-based chemi-resistor. A reliable solution for these drawbacks is the design and utilization of new gas sensitive materials based on hybrid inorganic–inorganic [8], organic–organic [12], and inorganic–organic materials [8, 9, 12, 16–36].

Using hybrid materials as sensitive transducer offers several obvious advantages, compared with the single constituent. First, the inexhaustible abundance of hybrid materials (in both the complex constituents and novel nanostructures) makes it possible to involve an almost infinite continuum of variable factors (surface-dependent factor, interface-dependent factor, and structure-dependent factor) to generate new sensing behaviors (Fig. 1c) [38–49]. Second, with hybrid material, more chemical/physical processes with different enhanced mechanisms could be introduced to precisely design, regulate, and enhance the sensing performance mainly through catalytic reaction with analyte [50–58], charge transfer [59–63], charge carrier transport [64–66] manipulation/construction of heterojunctions [39, 67], molecular binding/sieving [68–73], and their combinations [74–77].

## Hybrid Chemi-Resistive Gas Sensors

**Fig. 2:**
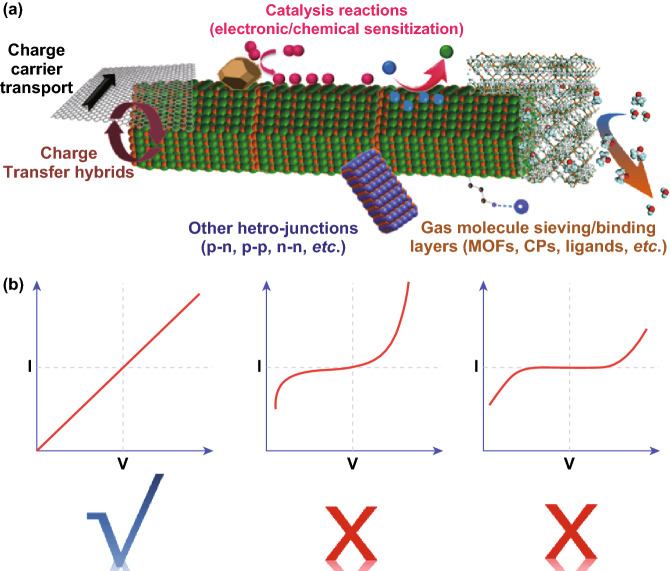
**a** Schematic illustration of five typical forms of hybrid functional nanomaterials for chemi-resistive gas sensing; **b** typical *I*–*V* curves of different sensors under DC bias (from left to right, chemi-resistors, chemical diodes, and proton/ions types)

Hybrid materials can perform improved sensing characteristics via one or a combination from five typical hybridizing forms which are categorized into three sensing-dependent factors (Fig. 2a). The first combination relies on catalysis reactions (normally noble metal catalysts, e.g., Pt [78], Pd [79], Au [50], and Ag [51]) between analyte gas and decorated catalysts on host semi-conductive materials (categorized as surface-dependent factor). The second relies on a fast charge transfer process, viz carrier withdrawal or donation, electron acceptor or acceptor between guest additives and the host material (e.g., carbon nanotubes (CNTs)), reduced graphene oxide (rGO) (categorized as interface-dependent factor) [63]. The third relies on regulating the charge carrier transport in a conductive/semi-conductive materials (e.g., single-wall carbon nanotube (SWCNT)-metallo-supramolecular polymer (MSP), gold nanoparticles (GNPs)-thiols, *N*,*N*’-diphenyl perylene tetracarboxylic diimide (PTCDI-Ph)/para-sexiphenyl (p-6P)) upon exposure to gas analytes (categorized as interface- and structure-dependent factor) [66, 80, 81]. The fourth relies on manipulation/construction of the heterojunctions such as n–n, p–n, p–p, p–n–p heterogeneous semi-conductive materials (categorized as interface-dependent factor) [39]. The last one relies on semiconductors coated by gas molecular sieving/binding layers or ligands/complexes for selective gas detection (categorized as surface- and structure-dependent factor) [72, 76, 82]. In the following section, we provide more details on each of these combinations. It should be noted that, according to the understanding of authors on chemi-resistors (1. measuring the resistors directly; 2. measuring the current when the device is applied a constant bias voltage; 3. measuring the partial voltage on the device in parallel with a constant resistance when the resistance and device is applied a constant bias voltage), we will review here only resistive-change sensing devices in which the contact of sensitive materials and electrodes is ohmic (good linearity of *I*–*V* curves under DC bias, Fig. 2b); therefore, we exclude devices in which the *I*–*V* curves under DC bias are nonlinear despite that they exhibit similar resistance changes (e.g., chemical diodes, proton/ions types).

### Hybrid Gas Sensors Based on Catalytic Effects

**Fig. 3:**
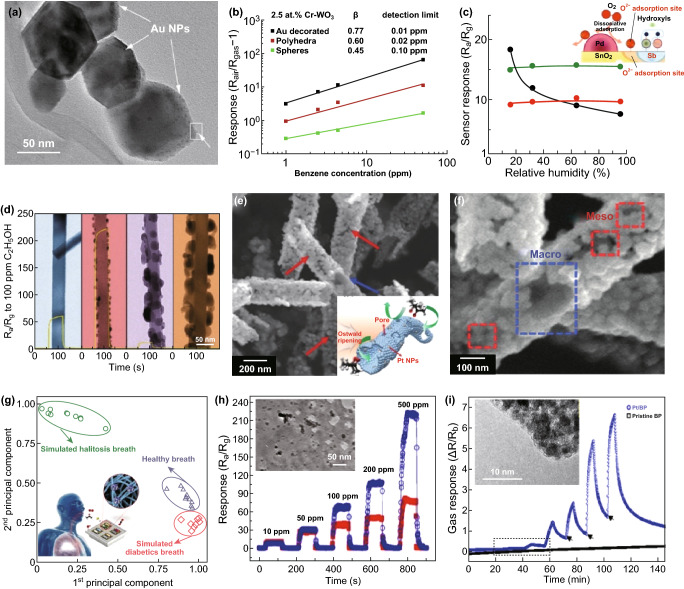
**a** SEM images of WO_3_ octahedron, **b** 2.5 at.% Cr–WO_3_ truncated octahedron and 10.0 at.% Cr–WO_3_ cuboid. Reproduced with permission [50]. Copyright 2015, American Chemical Society. **c** Humidity dependence of sensor response to 200 ppm hydrogen at 350 °C, using (black) undoped SnO_2_, (red) 0.1 mol % Sb-doped SnO_2_, and (green) 0.1 mol.% Pd-loaded and Sb-doped SnO_2_ nanoparticles. Reproduced with permission [89]. Copyright 2016, American Chemical Society. **d** TEM images of pure SnO_2_, 5Ag–SnO_2_, 10Ag–SnO_2_, and 50Ag–SnO_2_ NW after heating at 450 °C for 2 h and the corresponding response-recovery curves to ethanol gas. Reproduced with permission [51]. Copyright 2011, American Chemical Society. **e, f** SEM images of Pt–PS–SnO_2_ NTs (the inset is a schematic illustration). Reproduced with permission [52]. Copyright 2016, Wiley–VCH. **g** Pattern recognition by PCA using dataset from sensor arrays of PtM-decorated meso-WO_3_ NFs evaluating real and simulated (diabetes and halitosis) breath. Reproduced with permission [53]. Copyright 2017, Wiley–VCH. **h** TEM image of Pd nanoparticles@ZnO NSs and the corresponding response-recovery curves to acetone gas (red ZnO NSs). Reproduced with permission [54]. Copyright 2012, American Chemical Society. **i** Gas response of Pt/BP and pristine BP to various H_2_ concentrations (the inset shows the TEM images of Pt/BP). Reproduced with permission [55]. Copyright 2017, American Chemical Society. (Color figure online)

Catalytic effects of hybrid functional nanomaterials contribute to high response, fast speed, and low operating temperature via chemical/electronic sensitization, which is usually accompanied by synergistic effects, complementary behavior, and porous structures [50, 52, 83–86]. In addition, the exposed facets (morphologies) of matrix nanograins (facet-dependent chemical activity) and catalytic additives can greatly enhance the sensing properties of hybrid nanomaterials [87, 88]. For example, introduction of Cr dopants to WO_3_ polydedra can not only control the specific exposed facets and activation energy, but bring catalytic effects to the matrix [50]. The combining effects led to improved sensitivity and reduced operating temperature. Further hybridization with catalytic Au nanocrystals—to form Au/Cr–WO_3_ hybrids—contributed to high sensitivity, fast speed, and reduced working temperature to acetone and benzene due to Au/Cr co-catalysts-enhanced surface reaction (Fig. 3a, b). The advantages of co-catalysts can improve even further the hybrid materials. A recent example of this approach is Pd/Sb nanocrystals modification of SnO_2_ that Sb and Pd functioned as anti-humidity and catalytic sites, respectively, which remarkably reduced humidity interference and improved responses toward H_2_ (Fig. 3c) [89, 90].

The catalytic effects of loaded catalysts on host-sensitive materials are associated with the contact between catalyst and gas. A gas diffusion-favoured structure can provide additional exposed surface areas and fast speed via a combination of surface- and structure-dependent factors. The introduction of catalytic Ag NCs via *e*-beam evaporation and calcination into quasi-1D heterostructures significantly enhances the response and selectivity to ethanol (Fig. 3d) [51, 91]. For even further performance, quasi-1D nanostructures with both porosity and sensitive nanobuilding blocks, namely mesoporous 1D nanofibers/tubes (meso-NF/NTs), have been reported [41, 92–94]. By introducing sacrificial polymeric colloids and protein-templated catalysts to the solutions, meso- and macro-porous Pt-decorated SnO_2_ NTs have been fabricated by electrospinning and sintering in sequence (Fig. 3e, f) [52]. The combined effects of porous nanostructures, fully depleted sensing areas and uniformly distributed Pt nanocatalysts on SnO_2_ NTs allow a highly selective detection of acetone (R5ppm = 192). Similarly, bimetallic PtM (M = Pd, Rh, and Ni) catalysts can be introduced to meso-WO_3_ NFs that are then highly selective detectors of acetone and H_2_S gas [53]. Sensors array combined with pattern recognition methods (so-called e-nose) based on three different PtM-decorated meso-WO_3_ NFs can accurately detect and discriminate the breath of a simulated biomarker through principal component analysis (PCA, Fig. 3g).

**Fig. 4:**
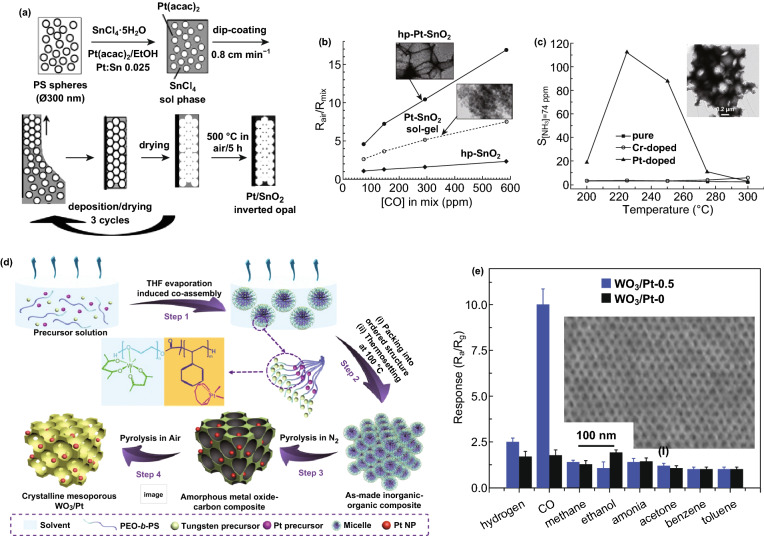
**a** Schema of the one-step preparation of Pt-doped SnO_2_-inverted opal films, and **b** responses comparison of different sensors as a function of CO concentration at 350 °C (insets are the corresponding HRTEM micrographs). Reproduced with permission [103]. Copyright 2010, American Chemical Society. **c** Responses comparison of different sensors as a function of working temperature to 74 ppm NH_3_ gas (the inset is the corresponding HRTEM micrograph of Pt-WO_3_-inverted opal films). Reproduced with permission [104]. Copyright 2011, American Chemical Society. **d** Synthesis of ordered mesoporous WO_3_/Pt hybrids. **e** Gas response of WO_3_/Pt-0.5 and WO_3_/Pt-0 to different gases (hydrogen, CO, methane, ethanol, ammonia, acetone, benzene, and toluene) at 100 ppm and 125 °C in 55–60% RH (the inset is an FESEM image of the crystalline WO_3_/Pt-0.5 viewed from the top surface). Reproduce with permission [56]. Copyright 2018, Wiley–VCH

**Fig. 5:**
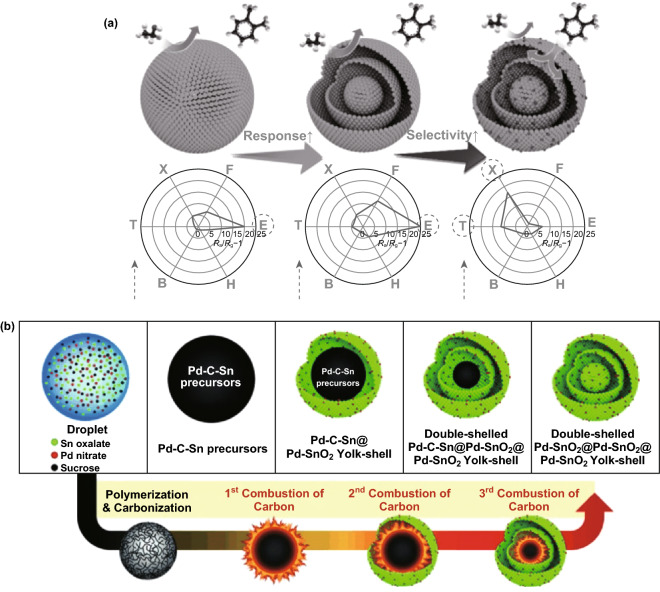
**a** Gas responses of dense SnO_2_ spheres, SnO_2_ yolk–shell spheres, and Pd-loaded SnO_2_ yolk–shell spheres to various analytical gases at 350-450 °C (B: benzene, H: H_2_, E: C_2_H_5_OH, F: HCHO, X: o-xylene, T: toluene). **b** Scheme showing the formation of double-shelled Pd-SnO_2_@Pd-SnO_2_@Pd-SnO_2_ yolk–shell spheres. Reproduced with permission [57]. Copyright 2014, The Royal Society of Chemistry

On the basis of intensive works on porous 2D ZnO nanostructures [37, 95, 96], Pd NCs have been deposited on porous 2D ZnO nanoplates (host materials) transformed by Zn_5_(CO_3_)_2_(OH)_6_ nanoplates to form 2D Pd/ZnO hybrid nanoplates (Fig. 3h); these acquire enhanced sensing properties [54]. Similarly, catalytic Pt can be used to decorate the surface of BP, which enables RT detection of H_2_ by Pt/BP at RT (Fig. 3i) [55]. When the structure of the host materials is further upgraded to 3D hierarchical porous (hp) nanostructures, a good gas diffusion platform is obtained with a large loading area of catalysts. By taking the advantage of the opals/polymeric beads or mesoporous silica/carbon/polymers templating method, hp-MOX thin films (3D hp nanostructures) with certain additives (catalysts) have been developed [97–102]. In this simple approach, hp-SnO_2_-inverted opal thin films loaded with mono-dispersed Pt catalyst (of uniform size of ~5 nm) were prepared (Fig. 4a) [103]. The improved sensing responses of Pt-doped SnO_2_-inverted opal thin films were achieved due to increased porosity, electronic sensitization, and synergism (Fig. 4b). Similarly, hp–Pt–WO_3_ or hp–Cr–WO_3_-inverted opal thin films have been successfully prepared (Fig. 4c) [104]. Due to the catalytic activation of N–H bond dissociation and effective gas diffusion within the macro-porous structures, excellent NH_3_ responses were obtained for the hp–Pt–WO_3_ sensor. As another example, well-controlled self-assembly of block copolymers such as poly(ethylene oxide)-blockpolystyrene (PEO–b–PS) could generate a perfect template with a highly ordered structure. Mesoporous WO_3_/Pt with a highly ordered and porous structure (inset of Fig. 4e) could be obtained by using this template and a 2-step pyrolysis process (Fig. 4d, including first treated in inert atmosphere and finally calcinated in air) [56]. Contributed to high surface areas (112–128 m^2^ g^−1^), large pore size (13 nm), and well-dispersed catalytic Pt NCs (~ 4 nm), the WO_3_/Pt-0.5 sensor had the highest response and fastest response-recovery speeds (Fig. 4e). Another category of nanostructures of host materials is multi-shell, yolk–shell, and multi-yolk–shell with hollow nanochamber [83–86]. With the hollow host nanochamber (SnO_2_) loading catalytic Pd through spray pyrolysis (Fig. 5a) [57], and showing the formation of double-shelled Pd–SnO_2_@Pd–SnO_2_@Pd–SnO_2_ yolk–shell spheres (Fig. 5b) [57], both sensitivity and selectivity were enhanced due to the unique hierarchical porous structure and uniformly exposed Pd catalysts. The representative works are summarized in Table 1.

**Table 1:** Representative works based on catalytic effects

Materials	Gas detection	Detection range	Work temperature	Refs.
WO_3_/Pt	CO	100–500 ppm	125 °C	[56]
SiO_2_/In_2_O_3_	NOx	970 ppb–97 ppm	RT	[220]
Pd/WO_3_	Acetone	50 ppb–500 ppm	300 °C	[221]
PdO/ZnFe_2_O_4_	Acetone	5–300 ppm	275 °C	[222]
Au/LaFeO_3_	Acetone	2.5–40 ppm	100 °C	[223]
Au NPs/ZnO	Athanol	5–60 ppm	RT	[224]
Sm_2_O_3_/SnO_2_	Acetone	0.1–200 ppm	250 °C	[225]
Pd/SWNT	CH_4_	6–100 ppm	RT	[226]
Pt/SnO_2_ NTs	CH_3_COCH_3_	10–100 ppb	350 °C	[52]
Pd@SnO_2_	H_2_S	5–100 ppm	290 °C	[227]
CuO/Pd	H_2_S	1–100 ppm	20-100 °C	[228]

As aforementioned, greatly improved sensitivity and speed have been realized by well design on the dispersion of catalysts/co-catalysts and gas diffusion-favored structures of hybrid gas sensors based on catalytic effects. The remaining problems of operating temperature and selectivity might be resolved by further combination with charge transfer (Sect. 3.2) and molecule sieving layers (Sect. 3.5), respectively. Moreover, the newly developed single atomic metal and/or metal cluster-based catalysts with better catalytic effect might bring new understandings and chance in such areas [105–108].

### Hybrid Gas Sensors Based on Charge Transfer Effects

**Fig. 6:**
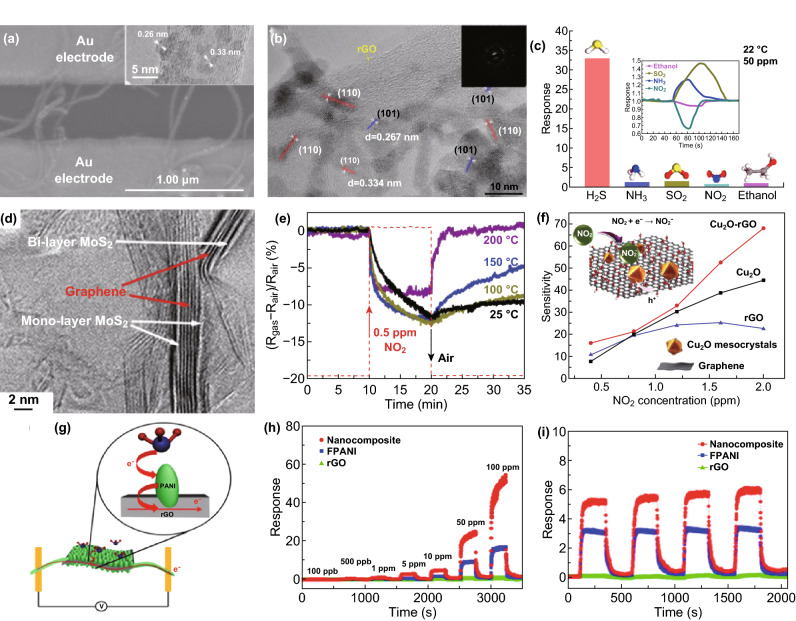
**a** SEM image of the sensor after assembly of SnO_2_ nanocrystals onto the MWCNTs (inset is the HRTEM image of a MWCNT uniformly coated with SnO_2_ nanocrystals). Reproduced with permission [59]. Copyright 2009, Wiley–VCH. **b** TEM image of SnO_2_ QDs/rGO hybrids; and **c** sensor responses to 50 ppm of different gas at RT. Reproduced with permission [109]. Copyright 2016, American Chemical Society. **d** Enlarged TEM image demonstrating the MoS_2_ coating of the few-layer graphene scaffold. **e** Sensor response to 0.5 ppm NO_2_ at different microheater temperatures, improving response and recovery time. Reproduced with permission [61]. Copyright 2016, Wiley–VCH. **f** Sensitivities of NO_2_ sensor for the three devices (inset is a schema of the mechanism of NO_2_ sensing of rGO-Cu_2_O). Reproduced with permission [62]. Copyright 2012, American Chemical Society. **g** Schema of the sensing mechanism of rGO-PANI hybrids. **h** NH_3_ responses of PANI, rGO and their hybrids at different concentrations. **i** Repeated NH_3_ responses of PANI, rGO and their hybrids at 10 ppm. Reproduced with permission [63]. Copyright 2016, Royal Society Chemistry

Charge transfer happens between decorations and the host materials (good conductivity), which could vary the conductivity of the hybrid materials. This process improves sensitivity to the analysts at low temperature or even at room temperature (RT), accompanying fast response and recovery properties [4]. Discrete and uniform SnO_2_ NCs-decorated multi-walled CNTs (MWCNTs) (Fig. 6a) [59] gave high performances (response of ~ 180% to 100 ppm of NO_2_) at RT due to the abundance of active sites and easy electron transfer under the assistance of the well-matched work functions of SnO_2_ and MWCNTs. Liu et al. [109] used rGO instead of the carbon nanotubes. This researcher successfully synthesized SnO_2_ QDs/rGO hybrids by a one-step solvent thermal reaction at 180 °C (oleic acid and oleylamine as capping agents) (Fig. 6b) [109]. Due to co-effects of excellent gas adsorption of QDs, effective charge transfer between SnO_2_–rGO interfaces and the superb transport capability of rGO, the sensor responses in 2 s with fully recovery properties upon exposure to 33 to 50 ppm of H_2_S at RT (Fig. 6c). Meanwhile, the SnO_2_/rGO-based sensor showed an obvious enhanced response compared with the responses of pure SnO_2_- or rGO-based sensors toward H_2_S at 22 °C, in which the rGO acted as a host transducer material. By combining both advantages, 2D MoS_2_ sheets were hybridized with 2D graphene to form rGO/MoS_2_ aerogel with large surface areas, porous structure, and high electrical conductivity (Fig. 6d) [61]. Efficient and rapid charge transfer across the interface ensured enhancement and fast detection of NO_2_ than bare rGO or MoS_2_ (Fig. 6e). Ascribing to the high specific surface area of porous Cu_2_O nanowires networks and improved conductivity via effective charge transfer, rGO–Cu_2_O mesocrystals had much higher sensitivity to NO_2_ at RT, surpassing the performance of stand-alone systems of Cu_2_O and rGO sheets (Fig. 6f) [62].

CPs could also be applied in charge transfer hybrids by replacing the inorganic components. For instance, graphene was combined with PANI to form a hybrid thin film that had improved, reversible, and stable NH_3_ sensing (Fig. 6g, h). The fast electron transfer between hybrids and NH_3_, assisted by π–π interactions of PANI and rGO with low electron transfer energy barrier, led to more electrons transfer from PANI to rGO; this effectively improved the responsivity and response time (Fig. 6i) [63]. Up to date, the detecting gases are limited to strong reducing/oxidizing molecules such as NO_2_, NH_3_, and H_2_S, which hinders the widely application of such hybrid gas sensors. The critical points to overcome this shortcoming may be depicted as: (1) the chemical/electronic modification of the reported charge transfer based hybrids to improve the sensitivity and expand the types of detectable gases (i.e., Pt–SnO_2_/rGO, details in Sect. 3.6); (2) the development on the new candidate of chemi-resistive decorations with desired absorption–desorption process and well-tailored energy level/energy band gap structure, which always shows low thermal activation energy (< 0.5 eV, i.e., electronic conductive metal–organic frameworks (EC-MOFs), for details see Sect. 3.5). The representative works are summarized in Table 2.

**Table 2:** Representative works based on charge transfer effects

Materials	Gas detection	Detection range	Work temperature	Refs.
CuxO/multilayer graphene	NO_*x*_	97 ppb–97 ppm	RT	[229]
rGO/NiO	NO_2_	0.25–60 ppm	RT	[230]
ZnO QDs/graphene	HCHO	25–100 ppm	RT	[231]
SnO_2_/rGO	H_2_S	10–100 ppm	RT	[109]
Graphite/polyaniline	NH_3_	50–1600 ppm	RT	[232]
SnO_2_/graphene	CH_4_	1000–10,000 ppm	150 °C	[233]
SnO_2_ CQD/MWCNT	H_2_S	3.3–100 ppm	70 °C	[234]
rGO/TiO_2_–Nb	CO	100–1000 ppm	380 °C	[235]
Fe_3_O_4_@RGO	NO_2_	50 ppb–50 ppm	RT	[236]

### Hybrid Gas Sensors Based on Regulation of Charge Transport

Different from charge transfer that simply uses high charge transport capability of the highly conductive component, hybrid gas sensors based on regulation of charge carrier transport can manipulate the sensing properties by changing carrier concentrations, transportation mode, and/or pathways of charge transport.

**Fig. 7:**
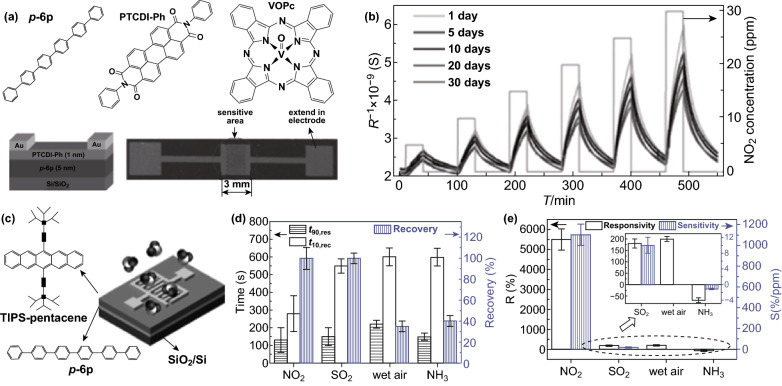
**a** Sensor device configuration and molecular structures of the materials. **b** The relative response of 1 nm PTCDI-Ph/5 nm p-6P film to NO_2_ pulses. The relative response curve is plotted as a function of time as the devices become exposed to different NO_2_ concentrations. Reproduced with permission [66]. Copyright 2013, Wiley–VCH. **c** Sensor device configuration and molecular structures of the materials. **d** The *t*_90,res_, *t*_10,rec_ and relative recovery after 10-min N_2_ pulse of the responses to different gases. **e** Responsivity (R) and sensitivity (S) to different gases. Reproduced with permission [64]. Copyright 2017, Wiley–VCH

**Fig. 8:**
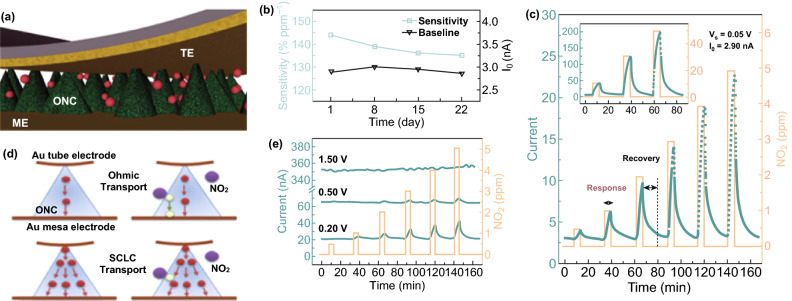
**a** Schema of the cross section of the tube/organic/mesa structure. VOPc nanopyramids (green cones) provide sufficient space for NO_2_ gas molecules to penetrate the structure. The red particles denote NO_2_ gas molecules. **b** Stability test in ambient atmosphere: sensitivity (left axis) and baseline (right axis) over time. **c** Response of periodic NO_2_ exposure. The device is operated under 0.05 V bias applied to the tube electrode. **d** Schematic diagram of charge transport and response to NO_2_ under ohmic transport (upper panel) and SCLC transport (lower panel) conditions. **e** Response of organic nanocrystal diode sensor (device 1) at 0.20, 0.50, and 1.50 V bias applied to the tube electrode. Reproduced with permission [65]. Copyright 2018, Wiley–VCH. (Color figure online)

A simple and effective way of enhancing responsivity relies on controlling the charge transport by tuning carrier concentrations. For example, a PTCDI-Ph/p-6P ultrathin film was fabricated with a thickness of only 6 nm, of which 5 nm was attributed to (p-6p/p type) asymmetric thickness and 1 nm to (PTCDI-Ph/*n* type) (Fig. 7a) [64]. Electrons in the PTCDI-Ph were deprived by NO_2_, which simultaneously released the restricted hole in p-6p, and thus influenced the transportation of p-6p; this generated a NO_2_ sensing signal at RT (Fig. 7b). Chi et al. [64] thermal-deposited a high-quality crystalline terrace-like TIPS-pentacene film on p-6P that can easily be positively charged (Fig. 7c). The efficient charge transport ability and low original carrier concentration gave superb NO_2_ sensing in terms of both response/recovery speed (Fig. 7d) and responsivity/sensitivity (Fig. 7e) [64]. Impressively, when the transport direction of charge carriers changes from horizontal to vertical in a vertical diode (containing top/down electrodes and VOPc/F16CuPc layers, Fig. 8a), the sensor responded remarkably well to 0.5–5 ppm NO_2_ at RT (Fig. 8c), with an acceptable sensing stability (Fig. 8b) and wide linear working region [65]. More interestingly, when the voltage bias is raised from 0.2 to 1.5 V, the sensing ability weakened dramatically (Fig. 8d), being ascribed to the transportation change from ohmic to space charge limited current (SCLC) mode (Fig. 8e) [65], which may give guidance as to how to choose the bias to control the charge transportation for gas sensors to get them to work under the best conditions.

**Fig. 9:**
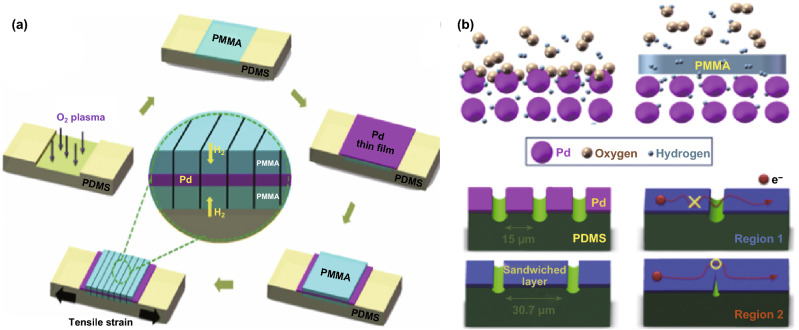
**a** Schematic diagram of the fabrication of nanogap sensors using PMMA/Pd/PMMA trilayer films on a PDMS substrate. **b** Schematic diagrams indicating the origins of the difference between the H_2_ detection limits of the Pd nanogap and the PMMA/Pd/PMMA hybrid nanogap sensors. Reproduced with permission [110]. Copyright 2014, Elsevier

**Fig. 10:**
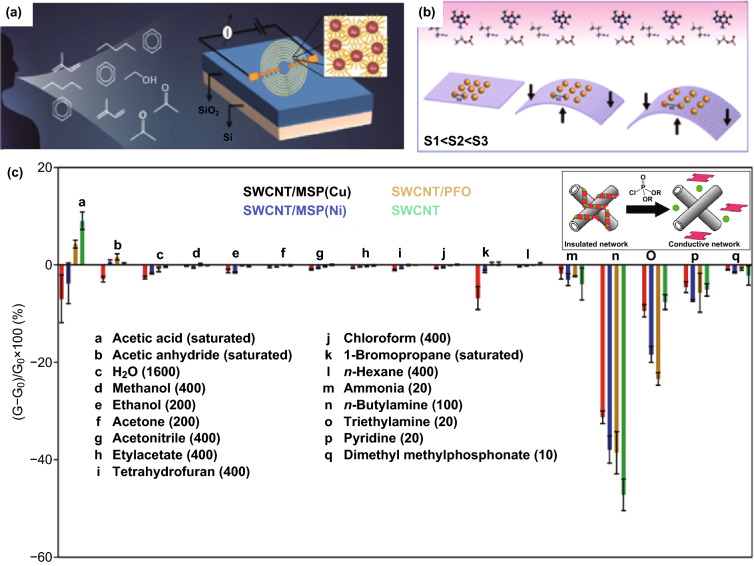
**a** Schema of the working principles of gold NPs thiol-coated thiols gas sensor. Reproduced with permission [81]. Copyright 2014, Elsevier. **b** Schema of a strain regulated gold NPs-based gas sensor. Reproduce with permission [112] . Copyright 2015, American Chemical Society. **c** Response of SWCNT-based chemi-resistive sensors after 50-s exposure to different vapors in N_2_ (concentration in ppm in parentheses). The inset is the schema of the working mechanism. Reproduced with permission [80]. Copyright 2015, American Chemical Society

Another strategy to manipulate the properties of sensors based on charge transport is by regulating the conductive pathways of conductive-insulate hybrid materials via physical cracks, chemical bindings, or the phase of the component. More specifically, this approach relies indeed on regulating the electron hopping barrier, the interspace between conductive materials, phase-change or their combination. Insulating polymers can be combined with sensitive inorganic materials to fabricate highly sensitive and selective chemi-resistors (Fig. 9a). Hybrid thin films as sandwiched PMMA/Pd/PMMA (PMMA = poly(methyl methacrylate)) were prepared on a flexible substrate using sputtering and spin coating in sequence (Fig. 9a) [110]. Hybrid thin films with nanogaps formed by 25% mechanical stretching have very selectivity and sensitivity in detecting H_2_ against O_2_, ascribed to the selective penetration of H_2_ in PMMA membranes and the density reduction of the cracks formed in the trilayer of the hybrid thin films (Fig. 9b). Adoption of similar principles, but with higher effects, relies on films of GNPs coated with monolayers of thiols [111]. These structures were a good solution for VOCs sensing due to the swelling and shrinkage of molecular chains interacting with VOCs (Fig. 10a) [81]. Assembly of the GNP-based chemi-resistors with a wide variety of functional groups creates sensor arrays with different resistances that can be further varied after interacting with VOCs [81]. The transport of electrons, expressed in electrical resistance, can be dually regulated by controlling the interspaces between GNPs after applying strain to the GNPs-based film, which further influence sensitivities (Fig. 10b) [112]. CNTs are also used as the host with the surface coverage of molecular (MSPs) (inset of Fig. 10c) [80]. Such MSPs could create sensory devices with a dosimetric (time- and concentration-integrated) increase in electrical conductivity triggered by electrophilic chemical substances (Fig. 10c).

In summary, for cases where the resistance decreased upon exposure to target gas, the depression of the off current via carrier concentrations reduction, transportation mode changes and/or physical cracks might be the most effective way to realize high sensitivity. Simultaneously, as presented above, the component used for the controlling charge transport of host materials can further contribute to improved speed, long-term stability, and excellent selectivity. The representative works are summarized in Table 3.

**Table 3:** Representative works based on regulation of charge transport

Materials	Gas detection	Detection range	Work temperature	Refs.
TIPS-pentacene	NO_2_	0.2–20 ppm	RT	[64]
PMMA/Pd/PMMA	H_2_	600–6000 ppm	RT	[110]
PANI/SWCNT	NH_3_	1–100 ppm	RT	[237]
Oleylamine/Pt	Organic contamination	< 0.3 ppm	RT	[238]
Ionic liquids/CNT	Heptanal	200 ppm	RT	[239]
	Toluene	1000 ppm		
	Ethanol	1000 ppm		
CNTs/hexa-peri-hexabenzocoronene bilayers	Decane	~ 10 ppb	RT	[240]
	Octane	~ 15 ppb		
	Hexane	~ 10 ppb		
	Ethanol	~ 10 ppb		
GNPs	Nonanal, styrene, ethanol, propionitrile	50–1000 ppb	RT	[112]

### Hybrid Gas Sensors Based on Heterojunctions

**Fig. 11:**
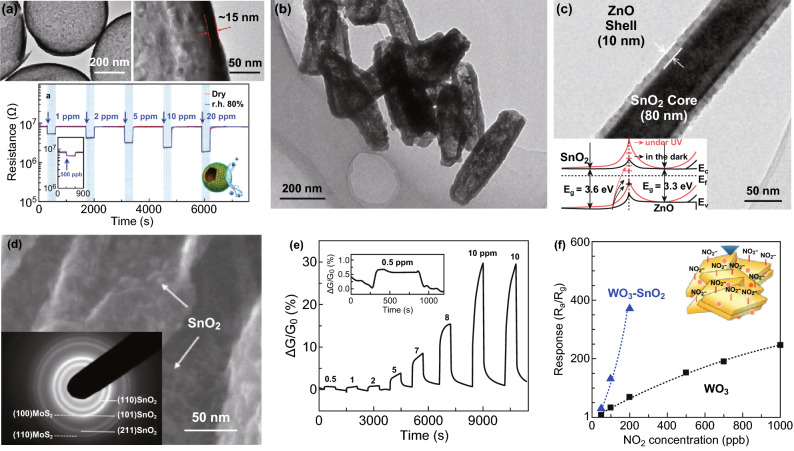
**a** TEM images and dynamic sensing transients of Ce–In_2_O_3_ hollow spheres exposed to 0.5–20 ppm of acetone at 450 °C in dry (red) and RH 80% (blue). Reproduced with permission [113]. Copyright 2016, Wiley–VCH. **b** TEM image of 1D Fe_2_O_3_/TiO_2_ tube-like nanostructures. Reproduced with permission [114]. Copyright 2012, American Chemical Society. **c** Low-magnification TEM image of SnO_2_/ZnO core–shell nanowires (the inset is the corresponding energy band diagram of the SnO_2_/ZnO system with/without UV light). Reproduced with permission [115]. Copyright 2013, American Chemical Society. **d** High-resolution SEM image showing that SnO_2_ NCs decorate on the MoS_2_ nanosheets (the inset is the corresponding SAED patterns), and **e** dynamic sensing response of the MoS_2_/SnO_2_ nanohybrids to different NO_2_ concentrations (the inset is the enlarged sensing response curve for 0.5 ppm NO_2_). Reproduced with permission [116]. Copyright 2012, Wiley–VCH. **f** Schematic of the sensing mechanism of WO_3_–SnO_2_ nanoplates and the corresponding responses comparison toward NO_2_ gas. Reproduced with permission [117]. Copyright 2014, American Chemical Society. (Color figure online)

Heterojunction is defined as the interface between two dissimilar semiconductors (one is the host, and the other one is the guest) that form a junction (n–n, p–p, p–n) linked with energy band structure due to the alignment of their fermi level. Notably, although the broad definition of heterojunctions covers all types of composites forming a junction in the interface, it is not clear enough for the well understanding of the complicated sensing mechanisms of composites sensing materials. Therefore, in this work, the narrow definition of manipulating/constructing heterojunctions is used, which excludes cases of catalytic effects, charge transfer, and charge carrier transport. According to the definition, the junction changes the interface potential energy barriers and regulates the transfer and/or injection of electrons and holes in a precise manner when it interacts with gas analytes. For example, n–n heterojunctions made of In_2_O_3_ hollow spheres (acetone-sensitive host) coated with CeO_2_ nanoparticles (humidity-sensitive guest) were synthesized and characterized as a chemi-resistive film. Exposing the layer to various gas analytes has shown selective detection of acetone in the presence of water, taking advantage of the chemical interaction between CeO_2_, In_2_O_3_, and water vapor, which greatly reduces the interfering effects of humidity (Fig. 11a) [113]. By modulating interface potential energy barrier between n–n junctions, as in the case of Fe_2_O_3_/TiO_2_ tube-like quasi-1D nanostructures synthesized through a multi-step hydrolysis (Ostwald ripening & thermal reduction), the corresponding sensing performance could be greatly improved (Fig. 11b) [114]. Combining modulation of electron transfer over the energy barrier at the perfect SnO_2_/ZnO heterojunction—fabricated by atomic layer deposition—and UV light generated electron–hole pairs, the sensitivity to NO_2_ could be improved using the SnO_2_/ZnO core-sheath nanowires (Fig. 11c) [115]. By introducing narrow band gap into the junction, such as in the case of In_2_O_3_ NCs to ZnO, a good response at visible-light conditions to gas analytes (e.g., formaldehyde) at RT (R100 ppm = 419%) [54] was attainable.

The unique morphology (good compatibility with the devices), nanoscale thickness, and high surface area of 2D nanostructures make them promising as the host materials for chemi-resistive gas sensors. Hybrids of SnO_2_ NCs-decorated MoS_2_ nanosheet (MoS_2_/SnO_2_) were synthesized via hydrolysis-pyrolysis processes (Fig. 11d) with air stability [116]. The SnO_2_ NCs not only enhanced the stability of MoS_2_ nanosheets in dry air, but served as strong dopants for MoS_2_, leading to the changes of conduction channels in the MoS_2_ nanosheets (Fig. 11e). For further improvements in the sensing performance, introduction of porosity, such as in the case of WO_3_ lamella-based films loaded with mono-dispersed SnO_2_ QDs (~ 4 nm) (Fig. 11f), could reach high levels [117]. Experimental results show that the porous lamellar-structured WO_3_–SnO_2_ hybrid films could achieve high response to NO_2_ gas, ascribing the effective insertion of QDs into lamella stacks as a strong electronic sensitization.

**Fig. 12:**
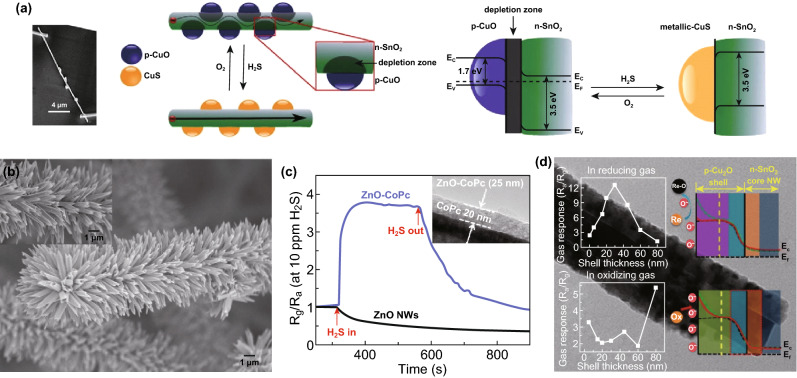
**a** SEM image of a single hybrid NW and the schematic illustration of gas-sensing mechanism of CuO–SnO_2_ p–n nanowires. Reproduced with permission [67]. Copyright 2013, Elsevier. **b** SEM images of the as-grown Te-coated SnO_2_ brush-like products prepared at source temperatures of 560 °C. Reproduced with permission [118]. Copyright 2014, American Chemical Society. **c** The dynamic response-recovery curves for ZnO and n-ZnO/p-CoPc to 10 ppm of H_2_S (the inset is the TEM image of n-ZnO/p-CoPc). Reproduced with permission [119]. Copyright 2015, American Chemical Society. **d** TEM image of 1D SnO_2_/CuO nanostructures (left insets are responses to reducing and oxidizing gas as a function of shell thickness, respectively; right insets are the corresponding sensing mechanism). Reproduced with permission [120]. Copyright 2015, American Chemical Society

Compared with n–n heterojunctions, p–n heterojunctions provide a stronger manipulation on interface potential energy barriers, build-in electric field and additional catalytic effects in some unique cases. For example, exposing p–CuO nanoparticles loaded on CuO–SnO_2_ p–n nanowires to H_2_S transformed it to highly conductive CuS (Fig. 12a) [67], resulting in depleted region change (the p–n junction breakup) and second-order effects (the oxidation of H_2_S by absorbed oxygen) after p-CuO was reversibly generated and removed on the SnO_2_ surface. Without generating new chemical compounds, simply tuning the thickness of in situ oxidation layer, rich Te–Te or TeO_2_/TeO_2_ bridging point contacts and additional p–n heterojunctions (Te/SnO_2_) contributed to further excellent sensing performances (to CO and NO_2_) of the brush-like heterostructures (Fig. 12b) [118]. The nanorods of *p*-type coating layer can be replaced by continuous layer to form core-sheath hybrids processing radial modulation of potential energy barriers, for instance, both n-ZnO/p-CoPc (cobalt phthalocyanines, Fig. 12c) [119] and n-SnO_2_/p-Cu_2_O (Fig. 12d) [120]. Core-sheath NRs have better sensitivity of the target gases.

**Fig. 13:**
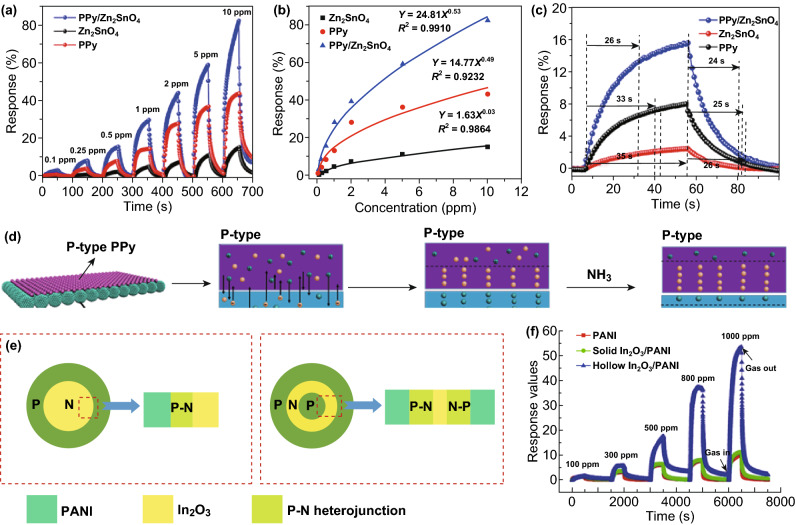
**a** NH_3_ response to PPy-Zn_2_SnO_4_, PPy, and Zn_2_SnO_4_. **b** Curve of concentration versus response. **c** Curve of a single response and recovery to NH_3_. **d** Schema of sensing mechanism. Reproduced with permission [121]. Copyright 2018, Elsevier. **e** Schema of sensing mechanism. **f** Gas response of PANI, solid In_2_O_3_/PANI and hollow In_2_O_3_/PANI. Reproduced with permission [122]. Copyright 2016, Springer

Conductive polymers (CPs, e.g., PPy, poly(3,4-ethylenedioxythiophene) (PEDOT), PANI) that are p-type components of diverse types of p-n heterojunctions can work at RT or low operating temperature with different working principles. First, PPy-ZnSnO4, p–n hybrid nanoparticles, can enhance the NH_3_ sensing performance (3–4 times higher) compared with pure PPy and ZnSnO_4_ (Fig. 13a) [121]. The concentration of NH_3_ can be quantitatively detected (Fig. 13b) with shorter time of response (26 s) and recovery (24 s) (Fig. 13c). The overall improved performance has been ascribed to the p–n heterojunction, in which the holes at high concentration in PPy and the electrons in Zn_2_SnO_4_ diffuse into each other to form a built-in electric field of a depletion layer (Fig. 13d). Interaction between Zn_2_SnO_4_–PPy and NH_3_ broadens the depletion layer, which determines the response, and the speed of response/recovery. When the p–n junction was reinforced as dual p–n junctions (p–n–p) in the hybrids of the hollow In_2_O_3_ nanofibers (NFs) and PANI (Fig. 13e), the performances were further enhanced (Fig. 13f) [122]. For CP-based chemi-resistive heterojunctions, unsatisfied sensitivity (response) and long-term stability will be two challenging issues for researchers.

Obvious advantages of heterojunction-type chemi-resistive hybrids-based gas sensor can be summarized as: (1) higher sensitivity due to manipulations of the potential energy barrier formed by band bending of different components (e.g., Fe_2_O_3_/TiO_2_ tube-like quasi-1D nanostructures (n–n) [114], n-ZnO/p-CoPc [119], and n-SnO_2_/p-Cu_2_O [120] core-sheath NRs); (2) improved selectivity to some gases (e.g., CuO–SnO_2_ p–n nanowires to H_2_S [67]); (3) promising, although limited so far, to anti-interference gas (e.g., CeO_2_–In_2_O_3_ hollow spheres with anti-humidity properties [113]); (4) avoiding UV-introduced ozone and performance degradation (for example, when narrow-band guest material hybrids with semi-conductive host materials, e.g., CdS-ZnO [123, 124], ZnO-CdS [125], CdSe-ZnO [126], the room operation temperature can be achieved by the visible-light-driven gas sensing). The representative works are summarized in Table 4.

**Table 4:** Representative works based on heterojunctions

Materials	Gas detection	Detection range	Work temperature	Refs.
α-Fe_2_O_3_/SnO_2_	Acetone	10–2000 ppm	250 °C	[241]
ZnO/SnO_2_	NO_2_	200–2000 ppb	RT	[242]
SnO_2_/SnS_2_	NO_2_	1–8 ppm	80 °C	[243]
SnO_2_/α-Fe_2_O_3_	Ethanol	20–100 ppm	225 °C	[244]
ZnO/ZnFe_2_O_4_	Acetone	5–700 ppm	250 °C	[245]
α-Fe_2_O_3_/NiO	Toluene	5–100 ppm	300 °C	[246]
SnO_2_/SnS_2_	NH_3_	10–500 ppm	RT	[247]
TiO_2_ QDs/NiO	NO_2_	5–60 ppm	RT	[248]
ZnO/MoS_2_	Acetone	10–500 ppb	350 °C	[249]
ZnO/ZnCo_2_O_4_	Acetone	50–300 ppm	175 °C	[250]
Si/SnO_2_	H_2_S	10–50 ppm	100 °C	[251]
SnO_2_@PANI	NH_3_	10 ppb–100 ppm	RT	[252]
NiO@SnO_2_	H_2_S	0.1–50 ppm	250 °C	[253]
SnS_2_/SnS	NO_2_	0.125–8 ppm	RT	[254]
SnO_2_/Sn_3_O_4_	NO_2_	20 ppb–50 ppm	150 °C	[255]
ZnO/ZnCo_2_O_4_	Acetone	10–100 ppm	275 °C	[256]
In_2_O_3_/ZnO	HCHO	5–100 ppm	RT	[257]

In summary, the critical points to achieve better performance in heterojunction-based gas sensor are depicted as: (1) maximum effective contact areas of the interfaces via surface and structure design; (2) matched band structure to facilitate the manipulation of potential energy barrier; (3) additional capability of catalysis of guest additives to host materials; (4) visible-light-driven photocatalytic abilities and good charge carriers separations for light-driven n–n or n–p hybrids; (5) selectivity-improvement-purposed heterojunction design based on specific interaction (e.g., Pd–H_2_, CuO–H_2_S, PPy–NH_3_) or n–p response-type reversion (Co_3_O_4_–SnO_2_ p–n junctions for H_2_) [127].

### Hybrid Gas Sensors Enhanced by Molecular Probing and Sieving Effect

Functionalization, coating or doping in/on the sensing materials, i.e., introduction of the sensing probe, was demonstrated as an effective way of improving the selectivity and specificity through a one-lock one-key binding or structure similarity-based combination. Both the inorganic and organic probes have been well developed. The sieving of interferon, especially the humidity in the environment, is an alternative way of improving specificity.

**Fig. 14:**
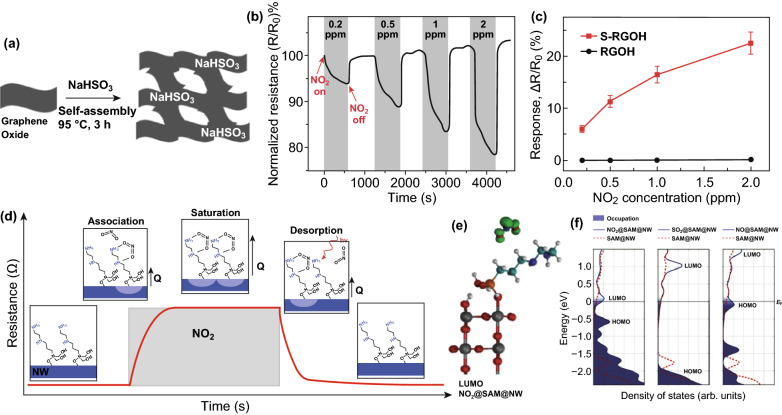
**a** Schema of the synthesis of 3D chemically modified graphene hydrogel. **b** Dynamic response of the 3D S-RGOH sensor versus time after exposure to 0.2–2 ppm NO_2_. **c** Plots of the quantitative responses of the S-RGOH and RGOH sensors versus NO_2_ concentration. Reproduced with permission [68]. Copyright 2017, Wiley–VCH. **d, e** Schematic illustration of the NO_2_ sensing mechanism by en-APTAS 1-modified NWs. **f** Density of states (DOS) of the en-APTAS 1-modified SnO_2_ with adsorbed NO_2_, SO_2_, and NO. The Fermi levels of the different systems are set at 0 eV. For comparison, the DOS of the en-APTAS 1-modified SnO_2_ NW without an adsorbed gas molecule (dashed red line) is shown in each graph. Reproduced with permission [70]. Copyright 2014, Wiley–VCH

**Fig. 15:**
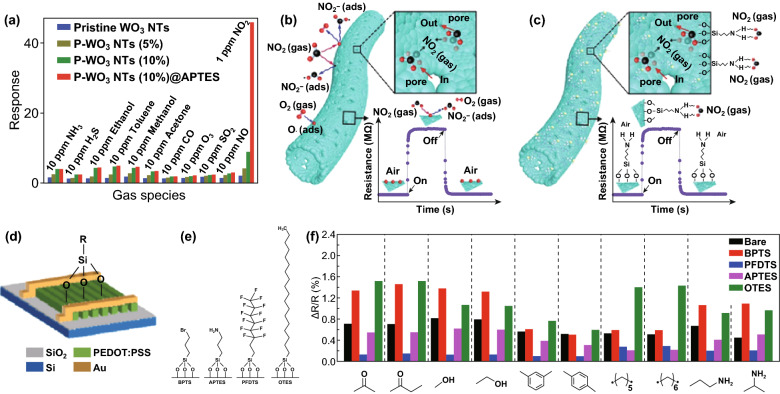
**a** Selectivity comparison of different gas sensors at the corresponding operating temperature. Schematic illustration of the NO_2_ sensing mechanism of sensors based on **b** P-WO_3_ NTs (10%) and **c** P-WO_3_ NTs (10%)@APTES. Reproduced with permission [71]. Copyright 2018, Royal Society of Chemistry. **d** The schema of silane-coated PEDOT:PSS sensor. **e** The molecular chain of different silane-coated on PEDOT:PSS. **f** Response to VOCs. Reproduced with permission [72]. Copyright 2018, Royal Society of Chemistry

A 3D sulfonated rGO hydrogel (S-RGOH)-based gas sensor combining chemical functionalization and porous structures was synthesized in a one-step hydrothermal reaction (Fig. 14a) [68]. Addition of a NaHSO_3_ probe dramatically enhanced the response (Fig. 14c) of NO_2_ with fast recovery (Fig. 14b), assisted by the porous structures of the graphene host. Self-assembled monolayers (SAMs) with suitable alignment of the gas–SAM frontier molecular orbitals (Fig. 14e) with respect to the SAM–NW Fermi level (Fig. 14f); this led to high selectivity and sensitivity to analyte gas [70]. SnO_2_ NWs were modified with amine-terminated SAM and applied as light-driven chemi-resistors working at RT, achieving good NO_2_ sensing performance, the schematic mechanism of which can be found in Fig. 14d. This concept was extended to porous MOX nanostructures for further enhancements of their sensing properties. APTES-modified porous WO_3_ nanotubes (P-WO_3_ NTs (10%)@APTES) performed the best sensitivity and selectivity (Fig. 14a), which can be ascribed to the large surface area and high gas diffusion rate provided by P-WO_3_, and selective reaction between NO_2_ and surface SAM with APTES (Fig. 15b, c). The existence of SAM on the surface of inorganic materials (except 2D nanomaterials) limits the working temperature, which greatly weakens the sensing performance, although it could be resolved by UV irradiation. Using conductive polymer as the host material with surface SAM functionalization by the “1-stone 2 birds” strategy was promising and novel (Fig. 15d, e) [72]. Superb sensing performances were achieved by combining RT sensitivity of CP and good selectivity of SAM (Fig. 15f) [72].

**Fig. 16:**
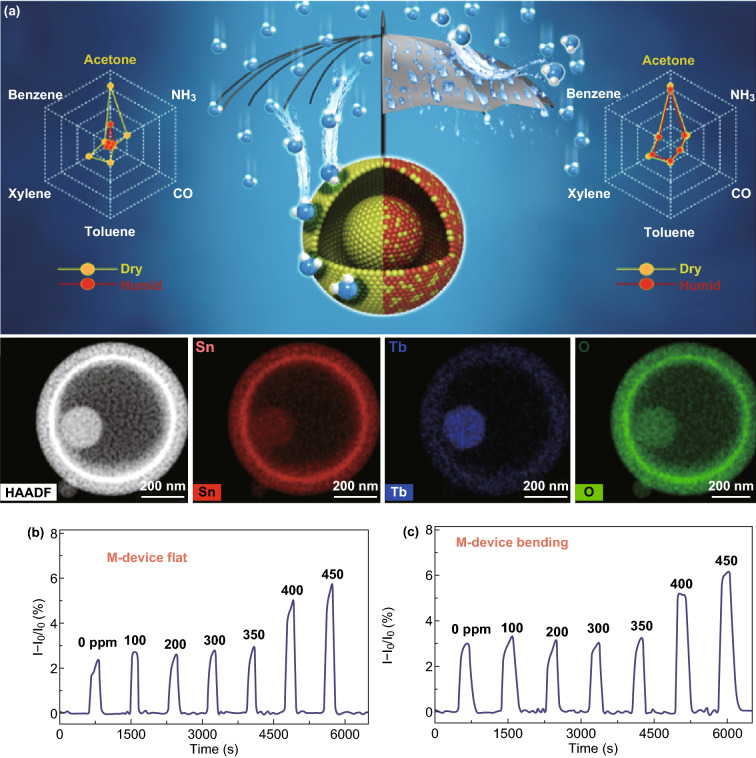
**a** Polar plots of the gas responses of pure SnO_2_ and 5 Tb–SnO_2_ sensors exposed to different gases (at 20 ppm) in dry (yellow) and RH 80% (red) atmospheres, and EDS elemental mapping of Sn, Tb, and O in 5 Tb–SnO_2_. Reproduced with permission [128]. Copyright 2018, American Chemical Society. **b, c** Response curves to 1-hexanal at different humidity in bending and flat states. Reproduced with permission [82]. Copyright 2018, Wiley–VCH. (Color figure online)

Figure 16a shows the low cross-sensitivity to humidity and other interferon gases by refreshing the regenerative surface involving the interaction between facile redox fair (Tb^3+^/Tb^4+^) and surface OH group (or water vapor) on SnO_2_. This 5 Tb–SnO_2_-based chemi-resistor achieved high response to acetone exposure [128]. The oleic acid SAM also was effective in screening the effect of humidity (< 350 ppm) when it was layered on PANI surface (Fig. 16b, c) [82]. Although it is not enough for practical application, this demonstration is still valuable in pointing to a promising way to eliminate the interferon of humidity.

**Fig. 17:**
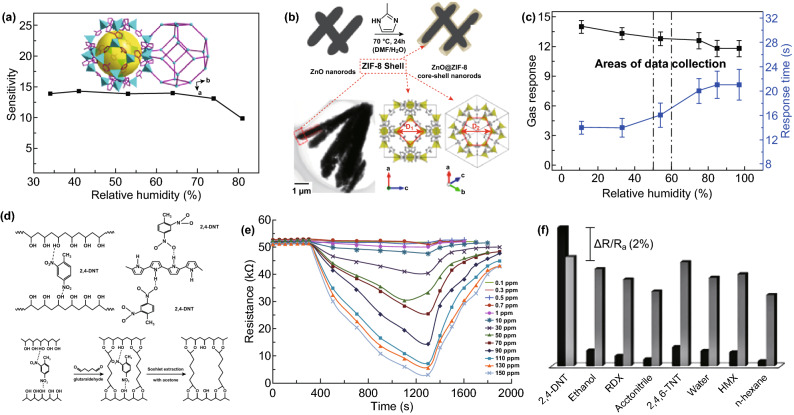
**a** Effect of environmental humidity on sensitivity of ZIF-67 sensor (inset is the SOD-type structure of ZIF-67). Reproduce with permission [129]. Copyright 2014, American Chemical Society. **b** Schematic diagram of the ZnO@ZIF-8 NRs synthesized with ZnO NRs as a template; and **c** gas response and response time for 100 ppm formaldehyde as a function of the relative humidity. Reproduced with permission [131]. Copyright 2016, American Chemical Society. **d** Schema of the interaction of 2,4-DNT with the hybrid ingredients (PVA, PPy, and MIP). **e** Response of a fabricated chemi-resistor sensor coated with PVA/PPy/MIP hybrids with respect to the 2,4- DNT explosive vapor. **f** Column curves of PVA/PPy/MIP hybrids to different organic compounds. Reproduced with permission [132]. Copyright 2018, Wiley–VCH

Recently, MOF materials are great opportunity in generating sub-nanometer or nanometer pores with high uniformity. Neat MOFs chemi-resistors were prepared based on hydrophobic MOF (ZIF-67), which showed selective response to VOCs, with slightly interfering effects of humidity (Fig. 17a) [129, 130]. ZnO@ZIF-8 core-sheath NRs powders were synthesized by hydrothermal reaction using a self-template strategy (Fig. 17b) [131]. The chemi-resistive gas sensor based on the thick film of ZnO@ZIF-8 hybrids had satisfactory sensitivity and response time to 100 ppm formaldehyde, even under interfering humidity (Fig. 17c). Mixing the CP with molecularly imprinted polymer (MIP) molecular (Fig. 17d) was another effective approach to improve not only the responsivity, but selectivity (Fig. 17f) [132]. Responses of interferon (2,4,6-TNT) and analyte (2,4-DNT) were suppressed and enhanced, respectively, although they are of very similar molecular structure and functional group [132]. However, the speed of response and recovery showed no obvious change (Fig. 17e) [132]. Instead of using functional MOFs as filter film coating on sensing materials to provide additional selectivity and/or sensitization, EC-MOFs are novel emerging materials with regular porosity and conductivity, which are promising for chemi-resistors with high sensitivity and selectivity [76, 133–158]. Unlike MOX and MOX-MOFs, which still need additional thermal or photonic energy as the trigger source to activate the sensing reaction, EC-MOFs can be directly used as sensitive materials based on their regular micro-porosity, selective frameworks, high electronic conductivity, and RT activity [139, 144–146, 159–166]. Therefore, EC-MOFs are promising components for hybrid gas sensors and will be powerful competitors for the new generation of gas sensors.

As mentioned above, the introduction of molecular probing and sieving effect can effectively overcome the poor selectivity problem of chemi-resistors. Up to date, only a few organic/inorganic probe or porous materials with molecule sieving effects have been applied to chemi-resistors to realize simple guest–matrix interaction (e.g., -NH_2_ group with NO_2_, –NO_2_ group with NH_3_, NaHSO_3_ with NO_2_) or molecule rejection (e.g., anti-humidity, gas molecules with large kinetic diameter). Such cases are ultra-small fraction of the state-of-art gas molecule adsorption and separation areas. More guest–matrix interactions (e.g., van der Waals interactions, hydrogen bond, π–π interactions, weak acid–base interactions) and gas separation design (e.g., channel traffic effects, framework flexibility), that have been well studied on rGO, polymers (e.g., metal-induced ordered microporous polymers (MMPs), covalent-organic frameworks (COFs), conjugated mesoporous/microporous polymers (CMPs)), MOFs, can be introduced to chemi-resistors for advanced sensing performances. The representative works are summarized in Table 5.

**Table 5:** Representative works based on molecular probing and sieving effect

Materials	Gas detection	Detection range	Work temperature	Refs.
ZnO@ZIF-71	Benzene	10–200 ppm	250 °C	[258]
ZnO@ZIF-CoZn	Acetone	0.25–100 ppm	260 °C	[259]
ZnO@ZIF-8	H_2_	5–50 ppm	250 °C	[260]
ZnO@ZIF-8	Propene Ethene	250 ppm	RT	[261]
Polyoxometalate @ZIF-8@ZnO	HCHO	25–200 ppm	RT	[262]
ZnO@ZIF-8	HCHO	10–200 ppm	300 °C	[263]
ZnO@ZIF-8	H_2_	10–50 ppm	300 °C	[264]

### Hybrid Gas Sensors Based on Combined Mechanisms

**Fig. 18:**
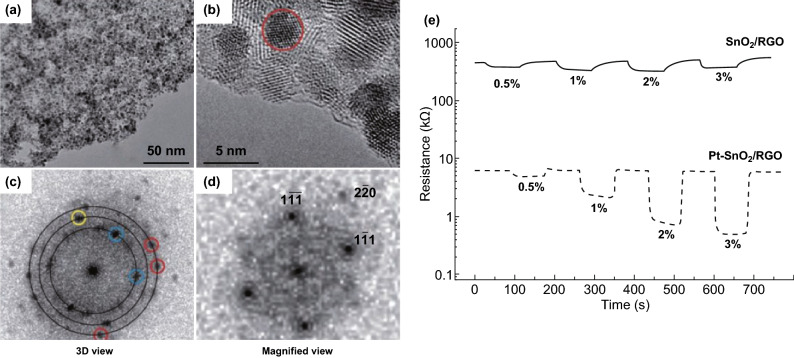
**a** TEM and **b** HRTEM images of Pt-SnO_2_/rGO. Power spectrum of **c** the whole HRTEM image and **d** the region of the HRTEM image indicated by the red circle. **e** Hydrogen-sensing performance at 50 °C of the materials under investigation: variation in the resistance of the rGO/SnO_2_ (solid line) and rGO/SnO_2_/Pt sensors (dashed line) with pulses of hydrogen at 0.5, 1, 2, and 3%. Reproduced with permission [74]. Copyright 2012, Wiley–VCH

**Fig. 19:**
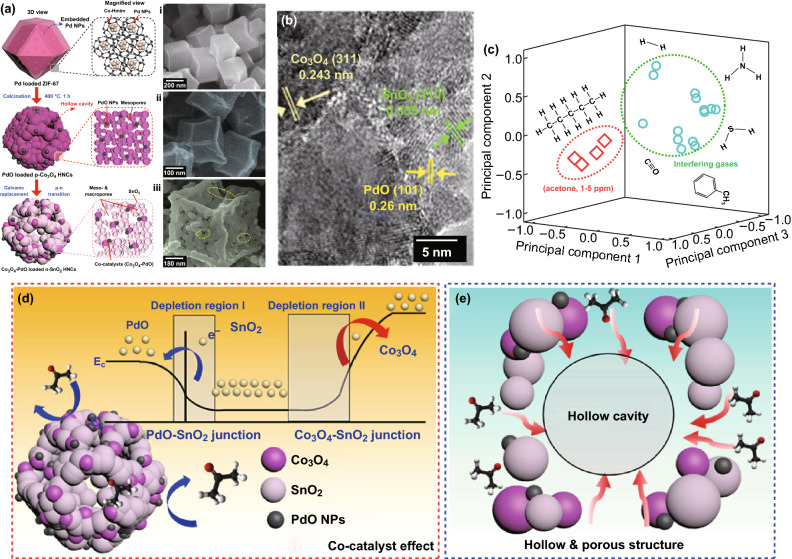
**a** Schematic illustration and SEM images of synthetic process for the n-SnO_2_ HNCs functionalized with Co_3_O_4_ and PdO. **b** HRTEM lattice spacing image of SnO_2_–PdO-loaded p-Co_3_O_4_ HNCs. **c** Pattern recognition based on PCA using sensor arrays (Co_3_O_4_-loaded n-SnO_2_ HNCs and Co_3_O_4_–PdO-loaded n-SnO_2_ HNCs). Schematic diagrams of **d** gas-sensing reaction derived from co-catalyst effect, and **e** magnified sensing mechanism on the porous surface of HNC. Reproduced with permission [75]. Copyright 2017, Wiley–VCH

In many cases, multi-forms working on the hybrid materials can simultaneously and dramatically improve sensitivity and responsivity. When the heterojunction barrier (the SnO_2_/rGO heterostructure interface) was combined with catalytic Pt, sensitivity of the SnO_2_/rGO hybrids to H_2_ was greatly enhanced (Fig. 18a–e) [74]. Hydrogen ranging from 0.5 to 3% in air could be quantitatively detected at near RT with response and recovery times of 3–7 and 2–6 s, respectively. Furthermore, when the catalytic effect was co-working with the p-n heterojunctions and porous structure (Fig. 19a), Co_3_O_4_–PdO loaded on n-SnO_2_ hybrid hollow nanocubes (Fig. 19b) reached selectivity and response amplitudes for the detection of acetone superior to those MOF-derived metal oxide sensing layers previously reported. Accordingly, the sensor arrays (Co_3_O_4_-loaded n-SnO_2_ HNCs and Co_3_O_4_–PdO-loaded n-SnO_2_ HNCs) can clearly distinguish 1 ppm humid acetone molecules among the seven interfering analytes (Fig. 19c). The reason is electron migration from n-SnO_2_ to PdO or p-Co_3_O_4_ in the multi-junctions significantly influencing the electron depletion regions, which leads to the superb sensitivity (Fig. 19d, e).

**Fig. 20:**
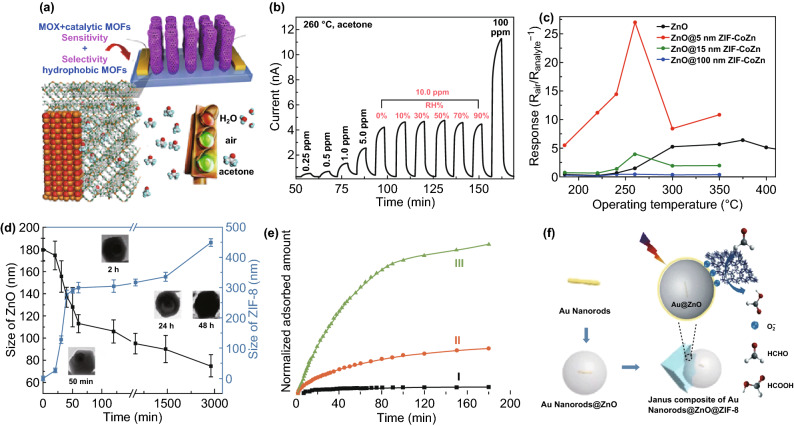
**a** Schematic illustration of ZnO@ZIF-CoZn core-sheath NWAs sensor; **b** Response-recovery curves to acetone at different concentrations in dry air and in 10 ppm acetone with different relative humidity and at 260 °C; and **c** temperature-dependent responses of ZnO@5 nm ZIF-CoZn. Reproduced with permission [76]. Copyright 2016, Wiley–VCH. **d** Plots of Au@ZnO and Au@ZnO@ZIF-8 size versus time; insets are the representative TEM images of the products at specific times. **e** Kinetics of HCHO adsorption (solid symbols). **f** Proposed mechanism of oxidation of HCHO into HCOOH. Reproduced with permission [77]. Copyright 2018, Springer

When the catalytic effect co-worked with the molecular sieving effect, improved selectivity (anti-interferon) with enhanced sensitivity can be achieved by coating a layer of hydrophobic and thermally catalytic bimetallic ZIF-CoZn thin film on ZnO to form core-sheath MOX@MOFs nanowire arrays (NWAs) (Fig. 20a) [76]. The bimetallic ZIF-CoZn MOF sheathes gave good thermal stability (ZIF-8(Zn)) and excellent thermal catalytic ability on ZnO (ZIF-67(Co)), as well as hydrophobic channels. By combining their advantages, the ZnO@ZIF-CoZn preparation showed greatly enhanced performance on selectivity (good anti-humidity, Fig. 20b) and also on its response, response and recovery behavior and working temperature (Fig. 20c). More complicated hybrid nanostructures containing MOX, plasmonic/catalytic NMs, and hydrophobic MOFs, i.e., the dual-functional Au@ZnO@ZIF-8 Janus structure (Fig. 20d, e), have been fabricated [77]. Au@ZnO@ZIF-8 hybrids had enhanced selective adsorption, detection and oxidation of HCHO and prevented interference from gases such as H_2_O and toluene (Fig. 20f), where Au NRs helped to generate charge carriers on a ZnO surface under visible-light irradiation. The representative works are summarized in Table 6.

**Table 6:** Representative works based on combined mechanisms

Materials	Gas detection	Detection range	Work temperature	Refs.
Co_3_O_4_/PEI-CNTs	CO	5–1000 ppm	RT	[265]
HC(NH_2_)_2_SnI_3_/SnO_2_/Pt	HCHO	5–100 ppm	80 °C	[266]
PdO/SnO_2_/CuO	CO	100–2000 ppm	200 °C	[267]
Pd/ZnO/In_2_O_3_	H_2_	50–172 ppb	350 °C	[268]
rGO/ZnO/Pd	CH_4_	25–500 ppm	RT	[269]
Pt/ZnO/g-C_3_N_4_	Ethanol	0.5–50 ppm	250 °C	[270]
	NO_2_	0.5–15 ppm	150 °C	
Au/Cu_2_O/ZnO	NO_2_	5–1000 ppb	RT	[271]
Ag/SnO_2_/rGO	Ethanol	100–2000 ppm	280 °C	[272]
TiO_2_/InVO_4_	NH_3_	10–1000 ppm	250 °C	[273]
Pd–SnO_2_/rGO	CH_4_	800–1200 ppm	RT	[274]
Au@In_2_O_3_@PANI	NH_3_	0.5–100 ppm	RT	[275]
Au–ZnO@ZIF-DMBIM	Acetone	0.0001–1000 ppm	RT	[203]
SnO_2_/α-Fe_2_O_3_/Pt	Styrene	0.25–1.25 ppm	206 °C	[276]

## Summary and Perspective

### Summary of Hybrid Gas-Sensitive Materials

The current progress report reviews advances and the advantages of the chemi-resistive hybrid nanomaterials compared with the single constituent, according to five main sensing mechanisms: manipulating/constructing heterojunctions, catalytic reaction, charge transfer, charge transport, molecular binding/sieving, and their combinations. Table 7 lists typical chemi-resistive materials for hybrid gas sensors categorized by types of materials and conductivity.

**Table 7:** Typical chemi-resistive materials for hybrid gas sensors

Types of chemi-resistive materials	Types of conductivity		
	*n*	*p*	*n*/*p*
Inorganic compounds	ZnO, SnO_2_,TiO_2_, MoO_3_, WO_3_, In_2_O_3_, V_2_O_5_, Ta_2_O_5_, Nb_2_O_5_, RuO_2_, MoS_2_, ZnSnO_3_	NiO, Co_3_O_4_, TeO_2_, CuS, Cr_2_O_3_, Sb_2_O_3_, CuO, Cu_2_O, Mn_2_O_3_, CeO_2_, PdO, Ag_2_O, Bi_2_O_3_, CoPc. WS_2_, MoSe_2_, LaFeO_3_ [277]	Fe_2_O_3_
Organic compounds	PTCDI-Ph	PPy, PEDOT, PANI, p-6P, Ti_3_C_2_T_x_ [278] PADS [279]	Polyphenylene [280]
MOFs	CuHITP	Cu-HHTP, NiHITP, NH_2_-UiO-66 [281],Cu-HHTP-THQ [204]	
Others			CNTs, BP, rGO

### Applications of Chemi-Resistive Sensor-Based e-nose

As the first commercial gas sensor, metal oxide-based chemi-resistors still occupy a leading role in both fundamental researches and commercial devices. Various commercial chemi-resistive gas sensors based on single or hybrid materials have been developed for the detection toward target gases (toxic, flammable, VOCs, explosive, H_2_, etc.) ranging from sub-ppm to saturated vapor, which are widely used in fields including environment monitoring, medical care, food industry, agriculture production, and public security. The versatile commercial chemi-resistive gas sensors are introduced but not limited as follows.

**Fig. 21:**
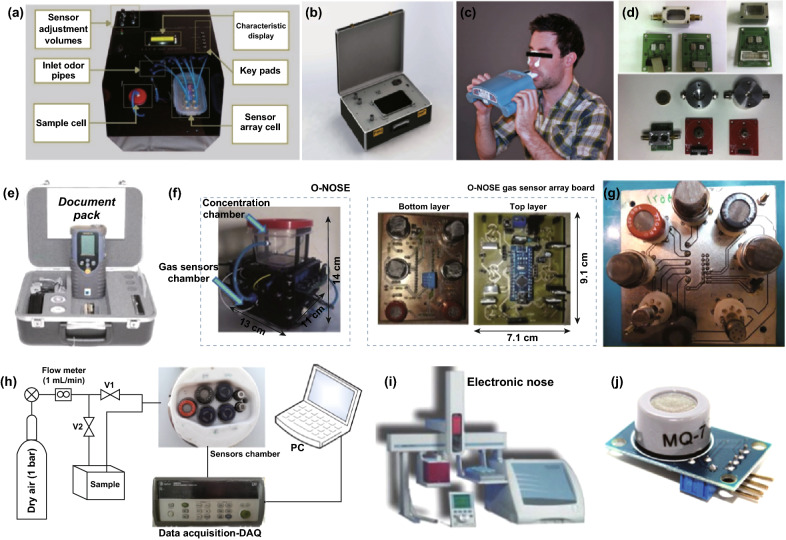
Schematic of the diffferent commercial e-nose. **a** Photographic image of Hanwei e-nose. Reproduced with permission [212]. Copyright 2017, Springer. **b** E-nose system “NOS.E” produced by Figaro Engineering Inc. [168]. Copyright 2018, IEEE. **c** Aeonose to diagnosis prostate cancer. Reproduced with permission [170]. Copyright 2018, European Association of Urology. **d** E-nose produced by institute of Physics Technology and Information, Spanish Council for Scientific Research. Reproduce with permission [213]. Copyright 2018, Elsevier Ltd. **e** The picture of Cyranose 320. Reproduce with permission [214]. From Chang and Heinemann, Copyright 2018, ASABE. **f** The sensors manufactured by Hanwei Sensors. Reproduced with permission [215]. Copyright 2019, Elsevier Ltd. **g** E-nose produced by Figaro Engineering, Inc., Hanwei company and FIS Inc. Reproduced with permission [216]. Copyright 2018, Elsevier Ltd. **h** E-nose based on MOS TGS and FIS sensors. Reproduced with permission [217]. Copyright 2018, sensors. **I** Fox 3000 electronic nose system. Reproduced with permission [218]. Copyright 2017, Elsevier Ltd. **j** MQ-7 (TORO) sensor model [219]

Some e-nose systems comprised of chemi-resistive sensor arrays have succeeded in the application of medical care. Commercial e-nose PEN3 (Airsense Analytics GmbH, Schwerin, Germany) made up of a gas sampling unit and a sensor array (10 different metal oxide thick film sensors (MOS)) can screen colorectal cancer (CRC) and polyps [167]. Another 14 commercial gas sensor-integrated e-nose system could generate characteristic “breath fingerprints” by exhalation components and could diagnose the lung disease through pattern recognition of a “breath fingerprint.” Those sensors categorized as MOS, hot wire gas, catalytic combustion gas, and electrochemical gas sensors are produced by the manufacturers, Hanwei (Fig. 21a), Figaro (Fig. 21b) [168], Winsen, Nemoto and Alphasense [169]. Aeonose in (Fig. 21c) [170] is a CE-certified, handheld, and battery-powered e-nose device designed by a Zutphen Company in Netherlands. The aeonose comprises three micro-hotplate metal oxide sensors and a pump to detect gastric cancer from exhaled breath [171]. Sunshine Haick Ltd. have successfully designed the sensor arrays to diagnose lung and gastric cancer via pattern analysis of exhaled VOCs, which has made great and has a perfect perspective. Other representative commercial e-nose in clinical diagnosis of complex regional pain, diabetics, head and neck cancer, dyskinesia, and prostate cancer are summarized in Table 8.

**Table 8:** Applications of electronic nose instruments for disease diagnosis

Diseases	Objective	E-nose configuration	Sensor type	Sensor arrays	Multivariate data analysis	Refs.
Armpit body odor	Detection and classification of human body odor	Tagushi (TGS) gas sensor from Figaro Engineering Inc.	MOS	5	PCA	[282]
Bile acid diarrhea (BAD)	Identify BAD in volatile organic compounds	The FOX 4000 e-nose from Alpha MOS, Toulouse, France	MOS	18	LDA	[283]
Lung cancer	Diagnosing lung cancer in exhaled breath	Cyranose 320 from Smiths Detection Inc., Edgewood, MD, USA	CP	32	LDA,KNN, PNN, NB, and SVM	[284]
Head/neck and lung carcinomas	Discriminating head and neck carcinoma from lung carcinoma	Aeonose from Zutphen, the Netherlands	MOS	3	PARAFAC and TUCKER	[285]
Prostate Cancer	The detection of prostate cancer from exhaled breath	Aeonose from Zutphen, the Netherlands	MOS	3	ANN	[170]
Complex Regional Pain	Diagnosing complex regional pain syndrome	Aeonose from Zutphen, the Netherlands	MOS	3	ANN	[286]
Mycobacterium tuberculosis	The detection of mycobacterium tuberculosis	ModelBH114-Bloodhound sensors from Leeds, UK	CP	14	PCA	[287]
Patients breath	The VOCs from breath	E-nose from Sunshine Haick medical Co.	GNPs capped with thiols	8–20	PCA, LDA	[112, 288]

*MOS* metal oxide semiconductor, *CP* conducting polymer, *GNPs* gold nanoparticles

Commercial e-nose has acted as an indispensable instrument for the rapid, accurate, and overall-process assessments of food health and quality aim at adulterated counterpart, contamination and spoilage [172]. An PEN-2 e-nose (WMA Airsense Analysentechnik GmbH, Schwerin, Germany) composed of 10 different metal oxide sensors was utilized to monitor the adulteration of milk with water or reconstituted milk powder [173]. Also, the PEN-2 is used to monitoring the change in volatile production of mandarin during different picking-date [174]. Meanwhile, PEN-2 is used to characterize espresso coffees brewed with different thermal profiles [13]. MOS sensors manufactured by Figaro (Figaro Inc., Japan) were used to recognize odors emitted from different stages in a waste water treatment plant [175]. Tagushi gas sensor based on metal oxide semiconductor from Figaro Engineering Inc. is used to classify the tea aroma [176]. Cyranose 320 in Fig. 21e that consists of an array of 32 thin-film carbon-black conducting polymer sensors was used to identify odor emitted from dairy operations. The portable e-nose based on thin-film semiconductor (SnO_2_) sensors (Hanwei Sensors) in Fig. 21f can perform early detection of wine spoilage thresholds in routine tasks of wine quality control. An e-nose system (Fig. 21g) was used to detect detergent powder in raw milk. Representative applications are summarized in Table 9.

**Table 9:** Applications of electronic nose for monitoring foods and beverage

Product	Objective	E-nose configuration	Sensor type	Sensor arrays	Multivariate data analysis	Refs.
Black tea	Monitoring of black tea fermentation process	Tagushi (TGS) gas sensor from Figaro Engineering Inc.	MOS	8	PCA, 2NM MDM	[289]
Pork	Measurement of total volatile basic nitrogen (TVB-N) in pork meat	Tagushi (TGS) gas sensor from Figaro Engineering Inc.	MOS	11	PCA and BP-ANN	[290]
Green tea	Identification of coumarin-enriched Japanese green teas and their particular flavor	E-nose device (FF-2A Fragrance & Flavor Analyzer, Shimadzu, Japan)	OSS	10	PCA, CA	[14]
Milk	Aroma profiling of milk adulteration	A PEN-2 e-nose from Win Muster Airsense Analytics Inc., Schwerin, Germany	MOS	10	PCA, LDA and 4ANN	[291]
Meat	Analysis adulteration of minced mutton with pork	A PEN-2 e-nose from Win Muster Airsense Analytics Inc., Schwerin, Germany	MOS	10	CDA, BDA, PLS, MLR, BPNN	[292]
Ham	Differentiation of hams marked	A PEN-2 e-nose from Win Muster Airsense Analytics Inc., Schwerin, Germany	MOS	10	PCA	[293]
Cherry tomato Juice	Classification with overripe tomato juice	A PEN-2 e-nose from Win Muster Airsense Analytics Inc., Schwerin, Germany	MOS	10	PCA, CA	[294]
Tea	Characterizing the degree of invasion of tea trees	A PEN-2 e-nose from Win Muster Airsense Analytics Inc., Schwerin, Germany	MOS	10	PCA and MLP	[295]
Sausage	Evaluation of lipid oxidation of Chinese-style sausage	A PEN-3 e-nose from Win Muster Airsense Analytics Inc., Schwerin, Germany	MOS	10	SVM, ANN, PLSDA, MLR	[296]
Mango	Quality rapid determination of mango	E-nose from HANWEI Electronics Co.	MOS	8	PCA and SR	[297]
Chicken fat	Rapid measuring of oxidized chicken fat	The FOX 4000 e-nose from Alpha MOS, Toulouse, France	MOS	18	APLSR and ANOVA	[298]
Honey	Identify the botanical origin of honeys	The FOX 3000 e-nose from Alpha MOS, Toulouse, France	MOS	18	PCA, DFA, LS-SVM and PLS	[299]
Orange juice	Classification of Valencia orange juices	The FOX 3000 e-nose from Alpha MOS, Toulouse, France	MOS	12	PCA, FDA	[300]
Wine	Geographical origin confirmation	The FOX 3000 e-nose from Alpha MOS, Toulouse, France	MOS	12	LDA	[301]
Coffee	Study the aromatic profile of espresso coffee as a function of the grinding grade and extraction time	αFOX from Alpha MOS, Toulouse, France	MOS	6	PCA	[302]
Cheese	Authenticity of cheese marked with Picorino	EOS 507 from Sacmi Imola S.C., Imola, Bologna, Italy	MOS	6	PCA, ANN	[303]
Tea	Classification of pure Sri Lanka tea	Model 3320 applied sensor lab emission analyzer from applied Sensor Co., Linkoping, Sweden	MOSFET	10	PCA, PLS	[304]
			MOS	12		
Honey	Botanical origin and adulteration with cane sugar	Cyranose 320 from Intelligent Optical Systems Inc., CA, USA	CP	32	PCA, LDA	[305]

*OSS* oxide semiconductor sensors, *MOSFET* metal oxide semiconductor field-effect transistor

The application of commercial e-nose to monitor volatile compounds in the environment both indoor and outdoor provides a reliable solution. Single semiconductor gas sensor GGS 10331 (produced by Umwelt Sensor Technik) was made with a semiconductor sensing layer on Al2O3 substrate to predict the concentration of ammonia under humidity interference [177]. Tagushi (TGS) gas sensor (Figaro Engineering Inc.) was applied to detect NH_3_, CO, H_2_, C_2_H_6_O, C_4_H_10_, C_3_H_8_, CH_4_, alcohol, and solvent vapors and the accuracy was 100% [178]. MQ-7 (Fig. 21j) is a commercial electronic nose for monitoring CO. Portable electronic noses in Fig. 21d were used to classify pollutants in water. Similarly, the commercial e-nose is widely used to identify the toxic wastes, soil/water pollution, indoor volatile organic compounds, etc. Table 10 summarizes the recent applications of e-nose for monitoring environment.

**Table 10:** Applications of electronic nose in environmental monitoring

Target gases	Objective	E-nose configuration	Sensor type	Sensor arrays	Multivariate data analysis	Refs.
Ethanol, acetone	The detection of ethanol and acetone in outdoor courtyard	Tagushi (TGS) gas sensor from Figaro Engineering Inc. (TGS2600, TGS2602,TGS2611TGS2620)	MOS	4	MoGC and K-NNC	[306]
Air	The quantification of VOC at indoor environments	Figaro TGS2602 air contaminant sensor	MOS	14	PLS-2 calibration models	[307]
Air	The detection of NH_3_, CO, H_2_, C_2_H_6_O, C_4_H_10_, C_3_H_8_, CH_4_, Alcohol	A sensor array composed of TGS826,TGS2442,TGS2600,TGS2602TGS2610,TGS2611,TGS2620	MOS	7	LDA, PCA, DT, KNN	[178]
Air	The detection of xylene	TGS2620	MOS	1	N/A	[308]
Air	The detection of n-hexane, acetone, and toluene	e-nose consists of TGS813, TGS2106, TGS2444, TGS244, TGS822, TGS2602, TGS2201, TGS2201	MOS	8	PLS and PCR	[309]
Air	The detection of methane, hydrogen, carbon monoxide	A gas e-nose system based on TGS2611, TGS821, TGS2442	MOS	3	ANNs	[310]
Inorganic analytes emissions	Analysis in landfill and industrial area chemical emissions monitoring	E-nose from Alphasense Inc.	MOS	7	LDA	[311]
Toxic wastes	The detection of ammonia	GGS10331 from Umwelt Sensor Technik	MOS	1	PCA and PLS	[177]
Air	The detection of NO_2_	MiCS-2714 produced by SGX Sensortech	MOS	1	N/A	[312]
Odorless gases	The detection of CO and CH_4_	MQ sensors from Hanwei Electronics Co.	MOS	6	ANN and LSR	[219]

E-nose is widely used in agricultural to analyze growth, classify seeds, detect the maturity, monitor quality, which promoted agricultural modernization and saved labor [2]. Eight MOS sensors produced by FIS (Osaka, Japan), MQ (Hanwei, China), and TGS (Figaro Engineering Inc.) were applied for classifying cumin, caraway, and other seeds [179]. Similarly, e-nose based on MOS TGS and FIS sensors (Fig. 21h) were distinguished Iranian Rosa damascena essential oils. An e-nose FOX 4000 (Alpha MOS, Toulouse, France) was chosen to analyze ginseng at different stages [180]. An e-nose FOX 3000 (Fig. 21i) was applied to characterize and classify seven Chinese robusta coffee cultivars. Commercially available chemical sensors intended for agriculture are summarized in Table 11.

**Table 11:** Applications of electronic nose in agriculture

Product	Objective	E-nose configuration	Sensor type	Sensor arrays	Multivariate data analysis	Refs.
Sesame oil	Detection adulteration in sesame oil	A PEN-2 e-nose from Win Muster Airsense Analytics Inc., Schwerin, Germany	MOS	10	PCA, FLT, Step-LDA, SFW, PNN, BPNN, GRNN	[313]
Olive oils	The detection of olive oils	A PEN-2 e-nose from Win Muster Airsense Analytics Inc., Schwerin, Germany	MOS	10	PCA	[314]
Red raspberries	The aromatic characteristics of red raspberries	A PEN-2 e-nose from Win Muster Airsense Analytics Inc., Schwerin, Germany	MOS	10	PCA	[315]
Compost maturity	The monitoring of composting process produces	A PEN-3 e-nose from Win Muster Airsense Analytics Inc., Schwerin, Germany	MOS	10	PCA	[316]
*Hyssopus officinalis*	Discriminate the accessions	A PEN-3 e-nose from Win Muster Airsense Analytics Inc., Schwerin, Germany	MOS	10	PCA and HCA	[317]
Rice	Estimation of the age and amount of brown rice plant	A PEN-3 e-nose from Win Muster Airsense Analytics Inc., Schwerin, Germany	MOS	10	PCA, LDA, PNN, and BPNN	[318]
Jujubes	Characterization of different varieties of Chinese jujubes	A PEN-3.5 e-nose from Win Muster Airsense Analytics Inc., Schwerin, Germany	MOS	10	PCA and LDA	[319]
Virgin olive oil	Adulteration with hazelnut oil	The FOX 4000 e-nose from Alpha MOS, Toulouse, France	MOS	18	PCA and PLS	[320]
Ginseng	Discrimination of American ginseng and Asian ginseng	The FOX 4000 e-nose from Alpha MOS, Toulouse, France	MOS	18	PCA and PLS	[321]
Flax seed oil	Differently processed oils for fraud detection	The FOX 3000 e-nose from Alpha MOS, Toulouse, France	MOS	18	PCA	[322]
*Lonicera japonica*	Quality control of Lonicera Japonica stored for different period of time	The FOX 3000 e-nose from Alpha MOS, Toulouse, France	MOS	12	LDA, PCA, and RBF-ANN	[323]
Tomato	Comparison of different stages of tomato	The FOX 4000 e-nose from Alpha MOS, Toulouse, France	MOS	18	PCA and ANOVA	[324]
White pepper	The chemical and flavor qualities of white pepper	α-Gemini from Alpha M.O.S. SA, Toulouse, France	MOS	6	PCA	[325]
Virgin olive oil	Confirmation of geographical origin and authentication	Model 3320 applied sensor lab emission analyzer from applied Sensor Co., Linkoping, Sweden	MOSFET	10	PCA, CP-ANN	[326]
			MOS	12		
Asphalt odor	Asphalt odor patterns in hot mix asphalt production	Cyranose 320 from Intelligent Optical Systems Inc., CA, USA	CP	32	Polar plots, PCA	[327]
Plant Pest and Disease	The discrimination of plant pest and disease	Model ST214 from Scensive Technologies Ltd., Normanton, UK	OCP	13	PCA, DFA, CA	[328]
Odors emissions	Monitoring of odors from a composting plan	EOS from Sacmi Group, Imola, Italy	MOS	6	kNN	[329]

*OCP* organic conducting polymer

Some commercial e-noses are attempted to detect explosives with ultra-low saturated concentration (parts-per-billion or below) [181]. Figaro Engineering Inc. produce an e-nose comprised of eight MOS sensors to discriminate and quantify different chemical warfare agents mimics [182]. More expectations in applications are also possible in the future.

### Challenges and Perspectives

Although excellent advances in both e-nose system and chemi-resistive sensory unit have been reached in the field during the last few years, there is still room for improvements. Below is a summary of the main rules for improving the performance of hybrid material-based gas sensors (details see the summary paragraph of each section):

For sensors based on heterojunctions (potential energy barrier manipulation), the more uniform and the larger the contact area of heterojunctions and charge transfer hybrids, the higher the response, resulting in faster speed/lower operating temperature, e.g., Fe_2_O_3_/TiO_2_ tube-like quasi-1D nanostructures (n–n) [114], n-ZnO/p-CoPc [119], CeO_2_–In_2_O_3_ hollow spheres with anti-humidity properties [113], and CdS–ZnO [123, 124].

For sensors based on catalytic effect assistance, the higher the potential energy barrier tuning, the higher the response, e.g., Pd/Sb–SnO_2_ [89, 90].

For sensors based on charge transfer, the more dispersion uniformity and the smaller size of catalysts on host-sensing materials, the higher the response, giving faster speed and lower operating temperatures, e.g., SnO_2_ QDs/rGO hybrids [109], rGO/MoS_2_ aerogel [61], and PANI/rGO [63].

For sensors based on regulation of charge carrier transport, the thinner and the more defect-rich of the hybrid film (e.g., suppression of original gas-off current in current-increased gas sensor), the higher and faster the responses obtained, e.g., PTCDI-Ph/p-6P ultrathin film [64], sandwiched PMMA/Pd/PMMA [110], and MSP-covered CNTs [80].

For sensors based on molecular binding/sieving, the more selective and uniform dispersion of molecular binding/sieving guests, the higher the selectivity, e.g., APTES-modified porous WO_3_ nanotubes [72], the oleic acid SAM-modified PANI [82], the ZnO@ZIF-CoZn NWAs [76] P-O3 NTs (10%)@APTES [71].

Improving the performance requires better understanding of the mechanism. Recently, most sensing mechanism represented in the research articles is “possible mechanism” based on the results of comparative tests instead of direct observations. Exactly, the latter one is more trustable and solid results to support the mechanism study, e.g., in situ FTIR [183, 184], in situ Kelvin probe [185], in situ STM [186, 187], in situ TEM [188, 189]). In addition, Theoretically studies (such as DFT simulation) [190], are also helpful for researchers to understand the interaction between the gas analyte and sensitive materials, the succedent electronic structure changes, or band gap regulation in heterojunction, or charge transfer, etc., which can guide the orientation of materials design [70, 190–195]. Otherwise, learning theoretical studies toward hybrid catalyst designs can inspire the further researches on hybrid gas sensing due to the similar surface physical/chemical science, band gap theories, and charge transfer process [196–201].

Controlling the fluidic behavior of gas [202], enhancing the anti-interferon ability by loading novel porous sieving materials (e.g., MOF, COF) [203–206], screening the cross-talk (such as deformation [207, 208]) by special micro-/nanostructures, deeply mining the features of sensing signal [209] (e.g., response, area of peak, and speed), and enhancing catalysis effect using small NPs, clusters, or even single-atom catalyst [108] are the long-term challenges of hybrid gas-sensing materials to adapt the applications under real-world conditions [210, 211]. The advances in knowledge in all our endeavors can be a foundation and useful experience for sensing technology, surface science, catalysis, fluidic mechanics, and microelectronics.
